# Peaking Global and G20 Countries’ CO_2_ Emissions under the Shared Socio-Economic Pathways

**DOI:** 10.3390/ijerph191711076

**Published:** 2022-09-04

**Authors:** Yuan Kong, Chao Feng, Liyang Guo

**Affiliations:** School of Economics and Business Administration, Chongqing University, Chongqing 400030, China

**Keywords:** carbon peak, STIRPAT, SSP, crowding-out effect, energy structure, energy intensity

## Abstract

Mitigating climate change requires long-term global efforts. The aim of this study is to simulate the possible paths of CO_2_ emissions in G20 countries and the world from 2020 to 2050, by using the STIRPAT model and SSP scenarios with different constraints (SSP baseline, SSP-3.4). The results show that: (1) the world’s CO_2_ emissions cannot peak in the SSP baseline scenarios, but can peak in the SSP-3.4 scenarios through four paths other than the high fossil energy consumption path; (2) for G20 countries, in the SSP baseline scenarios, 13 countries such as China, the United States, and the United Kingdom can achieve the peak, while six countries such as Argentina, India, and Saudi Arabia cannot. In the SSP-3.4 scenarios, Saudi Arabia cannot achieve the peak, while other countries can achieve the peak, and most of them are likely to achieve significant CO_2_ emission reductions by 2050; (3) climate goals have a crowding-out effect on other sustainable development goals, with less impact on developed countries and a greater impact on developing countries; and (4) the optimization of the energy structure and a low energy intensity can greatly advance the peak time of CO_2_ emissions.

## 1. Introduction

Since the Industrial Revolution, the world has experienced rapid development. Technological progress has led to social prosperity, along with reckless energy consumption, which has prompted a large number of greenhouse gas emissions, mainly CO_2_. According to the fifth report of the United Nations Intergovernmental Panel on Climate Change (IPCC), since 1850, the global cumulative anthropogenic CO_2_ emissions have shown a significant upward trend, and their concentration in the atmosphere has increased to an unprecedented level in the past 800,000 years [1]. This has broken the original carbon cycle balance and directly led to the consequences of global warming. According to statistics, human activities since industrialization have led to temperatures in recent years being about 1.0 °C higher than pre-industrial levels. Moreover, the global mean surface temperature (GMST) observed between 2006 and 2015 is already about 0.87 °C higher than the 1850–1900 average [2].

It cannot be ignored that the global temperature rise has subtly changed the climate conditions. Since 1950, many extreme weather and climate events have been observed, including the increase of extreme high temperature events and the increase of local heavy precipitation events [1]. In addition, natural disasters such as drought have gradually become difficult to predict [3]. In fact, as early as the 1990s, countries around the world have realized the seriousness of climate change caused by global warming. In 1992, 178 countries of the first United Nations Conference on Environment and Development reached an agreement and signed the United Nations Framework Convention on Climate Change. Since then, reducing carbon emissions to improve climate conditions has become a widespread issue around the world. It is undeniable that countries around the world have reached a consensus on climate change. In 2015, the Paris Agreement, reached by nearly 200 parties to the United Nations Framework Convention on Climate Change, established the goal of keeping the global average temperature increase well below the pre-industrial level of 2 °C, and striving to limit the temperature increase below the pre-industrial level of 1.5 °C [4].

In order to achieve the goals set by the Paris Agreement, some influential economies around the world have made emission reduction commitments in recent years, and some economies that are still in the stage of increasing carbon emissions have also given a timeline for the peak of carbon emissions. It is undeniable that the timing of carbon peak in some of the world’s major economies will be directly related to the success of controlling climate change. Therefore, predicting the carbon emission path has become a hot topic of current research. This can help us analyze the peak time of countries’ carbon emission and provide a basis for policymaking. This is also the main purpose of this paper. In addition, it is worth noting that the World Resources Institute (WRI) believes that the peak of carbon emissions does not only mean that carbon emissions peak at a certain point in time, but also must maintain a steady downward trend after the peak [5]. Therefore, although some studies have shown that some countries have reached the carbon peak [6,7], it is also of great practical significance to study the future emission paths of these countries. In view of this, this paper selects the G20 countries which have prominent influence on the world economy and carbon emissions as the research objects. Then, the future CO_2_ emission paths are explored by combining econometric analysis and scenario analysis. In addition, the world’s CO_2_ emissions will also be included in this study to investigate its future evolution path.

The rest of this paper is arranged as follows: Section 2 reviews the relevant research literature, which includes the current mainstream methods of carbon emissions research, especially the emission path prediction; Section 3 introduces the research methods and data sources of this paper; Section 4 reviews the history of CO_2_ emissions, then shows the results of the econometric and scenario analysis of the CO_2_ emissions path, and prospects for CO_2_ emissions in 2050; Section 5 summarizes the full text.

## 2. Literature Review

Since climate change caused by the greenhouse effect has attracted widespread attention, the discussion of greenhouse gas emissions-related topics, mainly CO_2_ emissions, has become the focus of academic research. For CO_2_ emissions, the current research mainly focuses on the discussion of its influencing factors [8,9,10,11,12,13]. On this basis, different types of research such as efficiency analysis and decoupling analysis are derived [14,15,16,17,18]. In addition to the analysis of influencing factors, another popular research idea is to predict the path of CO_2_ emissions. After a long period of development, the methods for predicting the path of CO_2_ emissions are rich enough to be self-contained. As the prediction of this paper is based on the econometric model to explore the influencing factors, this part will first introduce the literature on the influencing factors of CO_2_ emissions. Then, the method of predicting will be introduced.

For the research on the influencing factors of CO_2_ emissions, the main methods include structural decomposition analysis (SDA), index decomposition analysis (IDA), production decomposition analysis (PDA), and econometric research methods [9]. Taking the decomposition analysis method as an example, Wu et al. (2020) used the improved spatio-temporal structure decomposition analysis (ST-SDA), integrated the temporal and spatial dimensions, and explored the influencing factors of CO_2_ emissions with the data of Chinese provinces [12]. Chen et al. (2018) used the LMDI technique based on the IDA method to analyze the CO_2_ emission factors and their decoupling in OECD economies [8]. In addition, Zha et al. (2019) used IDA, PDA, and the Malmquist index to explore the influencing factors of CO_2_ emissions in China [13]. It is worth noting that articles using decomposition methods for analysis generally need to be based on the Kaya identity or IPAT framework, and rely on input–output analysis. Research using econometric methods also need to be based on the IPAT framework, but not on identities. Instead, they are based on the STIRPAT model with a random error term added. Based on this, econometric models can often consider more influencing factors, so they are widely used [9]. When using the STIRPAT model, various studies choose the total population, per capita GDP, and energy intensity as the main independent variables based on the IPAT theory. Other independent variables are selected according to the needs of the research topic.

With the rising voice of carbon emission reduction since the 1990s, the prediction of the CO_2_ emission path has also occupied an important position in many research directions. With regard to this kind of research, many current literatures combine various economic models and simulate the CO_2_ emission path through scenario analysis. In current studies, the analysis methods combined with scenario analysis include the LEAP model [19], grey model [20], system dynamics model [21], input–output decomposition model [22,23], econometric model, and so on [24,25]. Among them, the STIRPAT model based on econometrics and the LMDI method based on input–output have been widely used in the existing literature. They first examine the influencing factors of CO_2_ emissions and establish a prediction model based on it, and then explore the emission path combined with the future scenario. For the research based on the STIRPAT model, it is typical that Yuan et al. (2022) set scenarios for each Chinese province according to various economic and social planning documents, and used multiple regression to predict the peak value of CO_2_ emissions of Chinese provincial households [25]. For the input–output-based research, Hasan et al. (2020) used the LMDI method to decompose the CO_2_ emissions of the electricity sector in Bangladesh, and simulated the future CO_2_ emissions based on three scenarios [22]. As mentioned earlier in this section, the prediction method based on input–output is often likely to ignore the interference of random factors, so it has certain limitations in practical applications [9,26].

In view of the existing literature, the STIRPAT model, based on econometric analysis, can comprehensively examine the impact of population, economy, and technology on CO_2_ emissions, so this paper uses the STIRPAT model combined with the scenario analysis method to explore the path of CO_2_ emissions. The evolution of the STIRPAT model and the method of scenario analysis will be discussed in detail in the next section.

## 3. Methodology and Data

### 3.1. STIRPAT Model and PLS Regression

The model in this paper is based on the IPAT analysis framework. IPAT is an identity used to analyze environment-related problems, and its original form *I* = *PF* was set by Holdren and Ehrlich (1972) [27], where *I* is environmental pressure, *P* is population, and *F* is environmental pressure per capita. After that, Commoner developed it into the most classical form of *I* = *PAT* to explain the relationship between environmental pressure and population, wealth, and technology (1990) [28]. In view of the limited ability of IPAT equation to analyze the impact of social factors on the environment, *I* = *PACT* and other forms have been derived. However, none of them have solved the biggest limitations of the IPAT equation. First, the variables on both sides of the equation are simple linear relationships, and it is difficult to accurately measure the impact of changes in environmental drivers on environmental stress. Second, there is a lack of consideration of other random factors in environmental drivers. Third, hypothesis testing is not possible. To overcome these shortcomings, Dietz and Rosa built STIRPAT model based on IPAT framework (1994) [29]. The form of the model is:

(1) $$I=a\times P^{b}\times A^{c}\times T^{d}\times e\;$$

In STIRPAT model, *a* represents the constant, *b*, *c*, and *d* are the indices of population (*P*), wealth (*A*), and technology (*T*), respectively, and *e* is a random error term. In practical application, it is usually written as logarithmic form:

(2) lnI=a+b(lnP)+c(lnA)+d(lnT)+e 

The STIRPAT model allows more variables to be added and, therefore, provides a higher degree of flexibility in the analysis. On the premise of following the IPAT analysis framework, this paper takes CO_2_ emissions (*CE*) as the explained variable, and through a comprehensive review of the existing literature, five independent variables are added to the model as the driving factors. They are population (*P*), economic development level (*A*), energy intensity (*EI*), urbanization rate (*UR*), and the proportion of fossil energy to total energy consumption (*FS*). Among them, population (*P*), per capita GDP (*A*), and energy intensity (*EI*) can well represent population factor, wealth factor, and technology factor, respectively, of IPAT theoretical framework. The selection of urbanization rate (*UR*) and the proportion of fossil energy to total energy consumption (*FS*) in the STIRPAT model is for the completeness of variable selection. They are mainly used to measure the impact of population distribution and energy structure on CO_2_ emissions. In addition, in order to test the theory of environmental Kuznets curve, the quadratic term of economic development level is introduced into the expression. Thus, the extended STIRPAT model can be expressed as follows:

(3) $$\begin{array}{c} lnCE=lna+b_{0}\left(lnP\right)+b_{1}\left(lnA\right)+b_{2}{\left(lnA\right)}^{2}+b_{3}\left(lnEI\right)+b_{4}\left(lnUR\right)\\ +b_{5}\left(lnFS\right)+lne\; \end{array}$$

Considering the multicollinearity among the above variables (Table S1 in the Supplementary Materials) and referring to the research of Yuan et al. (2022), partial least squares regression is used for analysis in this paper [25]. Partial least squares regression is a technique which appeared in the 1980s. It can overcome the multicollinearity problem in multiple regression [30]. Through its algorithm, regression analysis, data structure simplification, and correlation analysis between two groups of variables can be realized simultaneously [31,32]. Since its birth, many algorithms have been derived, among which the nonlinear iterative partial least squares algorithm (NIPALS) has been the most widely used. This algorithm is used in this paper. It can be summarized as follows: *m* components *t* (*m* < *p*) are extracted from p standardized independent variables *E* by optimization iteration; *t* is a linear combination of *E*, which is pairwise orthogonal, carries the largest variation information in *E*, and has the largest correlation with the component *u* extracted from the standardized dependent variable *F*. After the iteration, the regression of *F* versus *t* is performed. Then, the regression equation is transformed into a non-standardized relationship by linear transformation [33].

In order to make the results more convincing, it is necessary to calculate the variable importance in projection (*VIP*) based on the regression results of PLS, so as to reflect the explanatory potential of independent variables to dependent variables [34]. A variable can be considered highly explanatory if it has a *VIP* value greater than 0.8 [35]. The formula for calculating *VIP* is as follows:

(4) $$VIP=\sqrt{\frac{p}{\sum_{h-1}^{m}r_{h}^{2}}\sum_{h-1}^{m}r_{h}^{2}w_{hj}^{2}}\;$$

where *p* is the number of independent variables, *w_hj_* is used to measure the marginal contribution of the *j_th_* independent variable to component *t_h_*, and *r_h_*^2^ is used to measure the explanatory power of the component *t_h_* to the dependent variable.

### 3.2. Scenario Analysis

In this paper, the method of scenario analysis is combined with equation (3) to investigate the future evolution of CO_2_ emissions at both country and global level. With regard to scenario analysis, there are many studies that rely on the government’s social and economic development planning to set the scenarios, which has obvious planning characteristics [19,20,21,23,25]. However, many drivers of CO_2_ emissions, in fact, have their own laws of growth. Relying too much on planning documents to set the scenarios will be unconvincing. Furthermore, it is inapplicable in the market-oriented environment of some countries. In addition to the scenarios drawn up by policy planning documents, Special Report on Emission Scenarios (SRES), Representative Concentration Pathways (RCP), and Shared Socio-economic Pathways (SSP) are also widely used for scenario setting in various studies [36,37,38,39,40,41]. Among them, SRES was developed in the early 1990s, which set four different possible future trajectories for population, economic growth, and greenhouse gas emissions. However, these scenarios are no longer applicable in the absence of some of the major changes that have taken place in society and the global economy in recent years. Since then, a team of researchers has developed RCP to describe possible future changes in radiative forcing caused by greenhouse gas emissions, which are reflected in the fifth report of the IPCC. However, its defect is that it does not set a matching socio-economic development scenario. In addition to RCP, another group of researchers developed SSP, whose baseline assumes no new climate policies. SSP sets out how various socioeconomic factors, including population, economic growth, urbanization, etc., will change in the future. In addition, SSP fully considers the growth rules of various socio-economic factors and can be used in combination with the climate change mitigation targets set by RCP [42].

**Figure 1:**
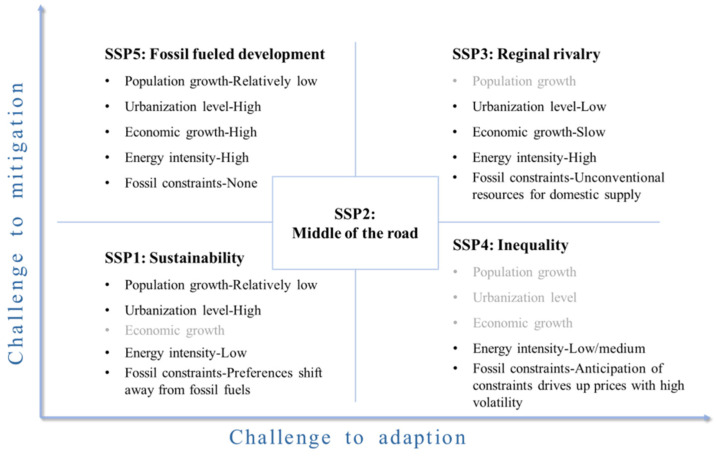
Baseline scenarios of shared socio-economic pathways.

SSP defines five baseline scenarios, a brief description of which is given below [43]. Additionally, Figure 1 summarizes the future scenarios for the variables selected in this paper.

(1) SSP1: A road of sustainable development. The challenges people face in mitigating and adapting to climate change will be relatively low. The world will develop inclusively and the ecological environment will be respected. Investment in education and health will accelerate the demographic transition. Countries will shift their focus from economic growth to human well-being. Inequality will be lower and consumption will be less energy-intensive.
(2) SSP2: Middle of the road. The challenges people face in mitigating and adapting to climate change will be modest. The world will follow a path in which social, economic, and technological trends do not deviate significantly from historical patterns.
(3) SSP3: A road of regional competition. People are facing great challenges in mitigating and adapting to climate change. Due to concerns about competitiveness and regional conflicts, the policies of various countries will gradually turn to face national and regional security issues. Due to excessive efforts in the face of security issues, countries will sacrifice broader development, including education investment decline, slow economic development, inequality deterioration, and serious environmental degradation.
(4) SSP4: A road of unequal development. The challenges people face in mitigating climate change will be low, and the challenge of adapting to climate change will be high. In this scenario, the inequality between and within countries in the world will become more and more serious. This will lead to a gradual increase in the development gap between different countries and different strata in the world. With the passage of time, there will be two extreme development situations, one is high-tech and capital-intensive, the other is the serious backwardness of social and economic development.
(5) SSP5: A road of high fossil fuel consumption. The challenges people face in mitigating climate change will be great, and the challenges of adapting to climate change will be relatively low. In this scenario, countries around the world believe in rapid development and technological progress, and the energy-intensive development model will promote rapid economic growth. In addition, people believe in the ability to manage societies and ecosystems, including through geoengineering if necessary.

The above presents the five SSP baseline scenarios from a qualitative perspective. For the quantification of SSP scenarios, IIASA summarized the values and growth rates of socio-economic factors in different regions in the future [44,45,46,47,48]. This paper selects the growth rate of each influencing factor to assign its future state. In addition to considering the CO_2_ emission paths under the five SSP baseline scenarios, this paper also sets scenarios (SSP-3.4) to limit the radiative forcing level to 3.4 W/m^2^ in 2100 by combining the SSP scenarios with the mitigation targets of RCP 3.4. It will help us get closer to the goal of 2 °C. Moreover, SSP-3.4 can provide us with a more realistic perspective before 2050 than the more stringent constraint of 2.6 W/m^2^ and the more relaxed constraint of 4.5 W/m^2^, which have been widely discussed. The detailed scenario settings are shown in Tables S3.1–20 in the Supplementary Materials.

### 3.3. Data

All variables selected in this paper are explained in detail in Table 1. The historical data of CO_2_ emissions, total primary energy consumption, and fossil energy consumption are from BP Statistical Yearbook. Among them, fossil energy consumption is the consumption of coal, oil, and natural gas in primary energy consumption. Population, per capita GDP and urbanization rate are from the official website of the World Bank. The selected data are from 1990 to 2019.

## 4. Results and Discussion

This chapter is divided into four parts to discuss. The first part reviews the CO_2_ emissions of G20 countries and the world in history, and then shows the regression results. The second part shows the projections of CO_2_ emissions in SSP baseline scenarios. The third part shows the projections of CO_2_ emissions in SSP-3.4 scenarios which include new climate policies. The fourth part looks forward to the CO_2_ emissions in 2050 and makes a comparative analysis with the emissions in 2019.

### 4.1. Historical CO_2_ Emissions

Table 2 shows the CO_2_ emissions of G20 countries and the world from 1990–2019. In addition to CO_2_ emissions, it also includes the proportion of individual CO_2_ emissions to the global total in 2019 and the average growth rate of CO_2_ emissions between 1990 and 2019. It can be seen from Table 2 that most of the major countries in the G20 are still in the rising stage of CO_2_ emissions, and only a few countries have achieved a steady decline in CO_2_ emissions from 1990–2019. In terms of individual CO_2_ emissions, China and the United States accounted for almost half of the world’s CO_2_ emissions in 2019. Combined with the rate of change, the United States has gradually achieved a steady decline in CO_2_ emissions, but China is not optimistic. In addition to China, two emerging economies, India and Indonesia, have also shown significant increases in emissions. In terms of the world’s CO_2_ emissions, they experienced a huge rise between 2000 and 2010, which were then eased after 2010, but it is still not optimistic.

In order to have a deeper understanding of the relationship between CO_2_ emissions and their driving factors, this paper strictly follows the IPAT framework. By reviewing the existing literature, population (*P*), economic development level (*A*) and its quadratic term, energy intensity (*EI*), urbanization rate (*UR*), and fossil energy share (*FS*) are added as independent variables in the STIRPAT model [8,9,10,11,12,13,25]. Through the variance inflation factor (*VIF*) test, as shown in Table S1, it is considered that the multicollinearity of the above variables cannot be ignored. In this paper, the PLS regression is chosen to solve this problem. Before using the PLS regression method, this paper first tests its applicability [49]. The t1/u1 scatter plot in Figure S2 shows a good correlation between the independent variable and the dependent variable after reconstruction. The t1/t2 scatter plot in Figure S1 shows that no outliers interfere with the regression. In Table S2, R^2^X (cum), R^2^Y (cum), and Q^2^X (cum) are all greater than 0.8, indicating that the model has good capabilities. In addition, the results of the jackknife hypothesis test for the standardized coefficients of each variable show that the regression results are valid [23,30,50].

In order to explore the importance of each variable in the data reconstruction, this paper calculates the variable importance in projection (*VIP*), which is shown in Figure S3. It can be seen from the figure that for all individuals studied in this paper, the proportion of fossil energy is not of high importance as an independent variable. In order to explore the role of each variable more clearly, Table 3 shows the non-standardized regression coefficients analyzed by PLS. Overall, population (*P*), economic development level (*A*), and fossil energy share (*FS*) are the main driving forces for the increase in CO_2_ emissions. Moreover, the regression results of economic factors suggest that there may be no inverted U-shaped relationship between per capita GDP and CO_2_ emissions. In terms of the above variables, different from the traditional Malthusian theory, the population of the United Kingdom and Germany shows a negative impact on CO_2_ emissions. This confirms Boserupian’s argument that population can reduce environmental stress by boosting science and technology [51,52]. In addition, for the variable of the urbanization rate (*UR*), different individuals show heterogeneity. The urbanization rate of some agriculturally developed countries shows a negative impact on CO_2_ emissions. For the variable of energy intensity (*EI*), mainly used to represent the technical level, except in Australia, the coefficients of all individuals are positive. This shows that the rise in the technological level is an important driving force to curb the increase in CO_2_ emissions. All these variables are internalized in SSP baseline scenarios and SSP-3.4 scenarios for future description.

### 4.2. Projections of CO_2_ Emissions in SSP Baseline Scenarios

**Figure 2:**
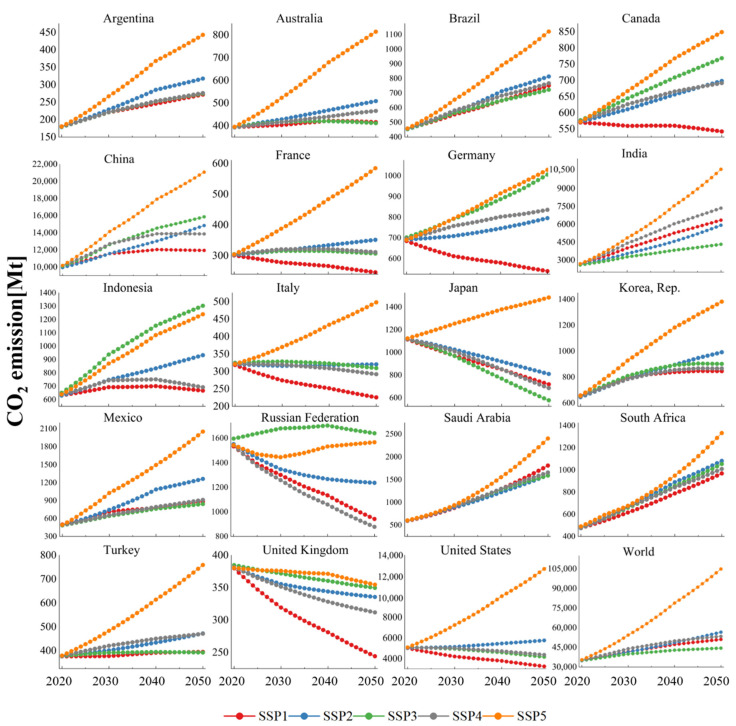
CO_2_ emission paths during 2020–2050 in baseline scenarios of SSP.

Combined with the regression results shown in Table 3 and the quantitative scenarios shown in Tables S3.1–20, Figure 2 shows the projected paths of CO_2_ emissions at both a country and global level from 2020 to 2050. The projected paths are based on the SSP baseline scenarios, which assume no change in climate policy. Obviously, for many countries where CO_2_ emissions are still growing, this is not a stringent requirement.

First of all, we analyze the CO_2_ emission trends and peaks at both country and global levels. From Figure 2, it can be seen that the world’s CO_2_ emissions cannot achieve the peak. In previous studies, Böhmelt also simulated the world’s CO_2_ emission path using the regression method combined with the SSP baseline scenarios. The results also show that the world cannot reach the peak of CO_2_ emissions in 2050 [53]. In contrast to Böhmelt’s research, this paper enriches the model variables and uses the latest SSP database. Therefore, the CO_2_ emissions under different SSP baseline scenarios have been reordered. It can be seen from the Figure 2 that the world’s CO_2_ emissions are in an upward trend in all paths, with the largest increase in the SSP5 scenario and the smallest increase in the SSP3 scenario. Of all countries, the United Kingdom performs best, with a steady decline in CO_2_ emissions across all five baseline scenarios. In addition, France, Italy, Japan, the Russian Federation, and the United States also perform well in some scenarios, and their CO_2_ emissions show a decline in at least three paths. It is worth noting that in the countries studied in this paper, except China and India, there has been at least one peak of CO_2_ emissions in 1990–2019. However, from Figure 2, some countries’ CO_2_ emissions in the future will perform badly, mainly for some emerging economies and developing countries. Some of these countries will not be able to peak again even in all baseline scenarios by 2050. This shows that it is not easy to strike a balance between development and CO_2_ reduction. It is worth noting that there has been a lot of discussion at climate summits on how to help developing countries reduce CO_2_ emissions. However, it is not enough for us to rely solely on existing climate policies or climate-related agreements to achieve emission reduction goals.

For the single scenario:

(1) According to the SSP1 baseline scenario, the world will generally change its existing development path and optimize it in a sustainable direction. Due to a relatively low population (*P*) change, low energy intensity (*EI*), and gradually decreasing fossil energy share (*FS*), it can be found that in the SSP1 scenario, at least half of the countries can reach the peak and enter a steady downward trend in the future, including China, which currently contributes the most to global CO_2_ emissions. The emission path of the world in the SSP1 scenario is also at a low level. This suggests that a sustainable development path is feasible for reducing CO_2_ emissions when there is no new climate policy.
(2) In the SSP2 baseline scenario, countries around the world will almost maintain their existing development patterns. Combined with Figure 2, it can be found that this route is not conducive to reducing CO_2_ emissions. Only Japan, the Russian Federation, and the United Kingdom can enter a stable downward trend of CO_2_ emissions. Combined with the actual situation, there are still developing countries such as India in the extensive model of development, and the transformation of the development model cannot be achieved overnight. In addition, this result also shows that some countries which have achieved the peak of CO_2_ emissions in history still need to further optimize their development model, otherwise they may not achieve the real ‘carbon peak’.
(3) In the SSP3 baseline scenario, countries will embark on a road that focuses on regional security and high-intensity competition, which will greatly weaken the development potential of some emerging economies and developing countries. In this scenario, the level of economic development is generally lower than that set in other scenarios. This will make the world’s CO_2_ emissions path reach the lowest level in all baseline paths. It is clear that this competitive scenario will inevitably lead to unequal development at an international level and will not be conducive to the prosperity of the world. This is not in line with the consensus reached by most countries in the United Nations’ Sustainable Development Goals (SDGs).
(4) In the SSP4 baseline scenario, the world will move towards an unequal development model, including inequality at an international level and inequality within countries or regions. This scenario corresponds to a relatively low energy intensity (*EI*), while population (*P*), economic development level (*A*), the urbanization rate (*UR*), and fossil energy share (*FS*) are set separately based on the situation of each country. Combined with Figure 2, we find that the CO_2_ emission path under the SSP4 setting, which represents inequality, is unexpected because it can achieve relatively low CO_2_ emissions in most of the countries. There is a lot of discussion about the impact of inequality on CO_2_ emissions in various studies. If we use income inequality to measure development inequality, some studies suggest that the relationship between income inequality and CO_2_ emissions shows different signs between rich countries and low-income countries [54,55]. In addition, some studies show that high-income groups within a country have a more positive effect on reducing CO_2_ emissions [56].
(5) In the SSP5 baseline scenario, countries will pay less attention to energy structure optimization and energy saving. This will lead to a high proportion of fossil energy (*FS*) and a high energy intensity (*EI*). It is worth noting that the setting of the population (*P*), economic development level (*A*), and urbanization rate (*UR*) in SSP5 is close to that in SSP1. Moreover, in the SSP5 scenario setting, it is considered that countries will take environmental measures other than energy structure optimization to reduce CO_2_ emissions. However, it is clear whether, under such a scenario, the world would be on a path to irreversible climate change. Except for the United Kingdom, which can maintain a downward trend of CO_2_ in this scenario, other countries will maintain a relatively divergent emission path. From the perspective of the world’s CO_2_ emission path, the path under the SSP5 baseline scenario is much higher than that under other scenarios, and there is no convergence trend in this path, which implies that all the efforts made by the world to change climate change may be in vain.

In general, according to the emission paths in all SSP baseline scenarios, countries in the world should avoid a path of high fossil energy consumption and high energy intensity in any case. Among other development models, although the SSP3 scenario has the most positive effect on the world’s overall CO_2_ emission reduction, it is not in line with the realization of common prosperity worldwide. Therefore, under the condition of maintaining the existing climate policies, the sustainable development scenario corresponding to SSP1 is undoubtedly the best setting.

### 4.3. Projections of CO_2_ Emissions in SSP-3.4 Scenarios

**Figure 3:**
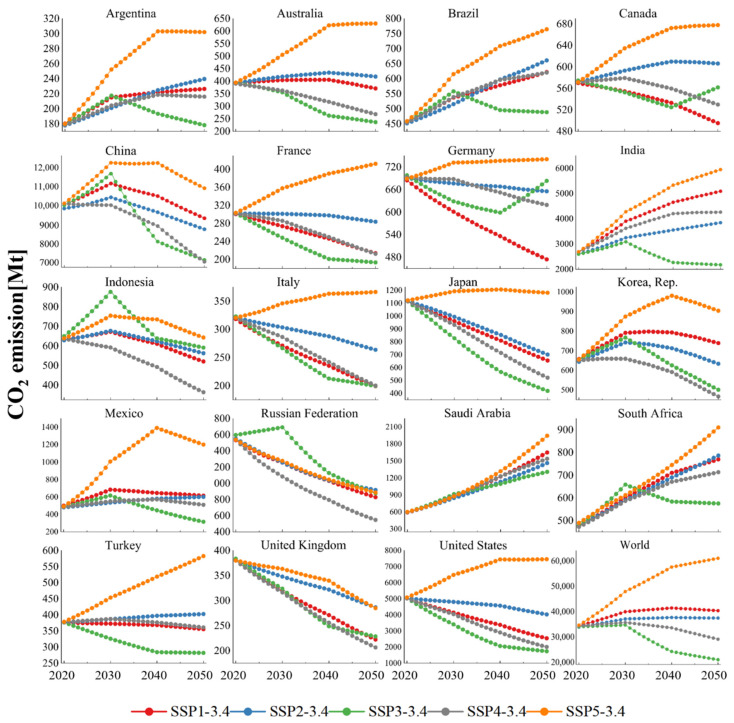
CO_2_ emission paths during 2020–2050 in 3.4 W/m^2^ scenarios of SSP.

Figure 3 shows the paths of CO_2_ emissions for each SSP scenario with new climate policies. The new climate policies aim to limit the radiative forcing to 3.4 W/m^2^ in 2100, which is more stringent than the baseline scenarios. It can be seen from Figure 3 that the paths corresponding to the SSP-3.4 scenarios have a lower CO_2_ emission level than the paths corresponding to the SSP baseline scenarios. In SSP-3.4 scenarios, only Saudi Arabia cannot reach the peak. Most countries can peak the CO_2_ emissions on at least three paths, including some emerging economies and developing countries that are difficult to peak in the baseline scenarios. It should be noted that compared with the baseline scenarios, the SSP-3.4 scenarios only make changes that can represent the energy structure, technology level and development mode, which are reflected in the changes in the proportion of fossil energy (*FS*) and energy intensity (*EI*). Therefore, it can be considered that there is a theoretical possibility of reducing CO_2_ emissions without affecting economic development. However, to make this possibility a reality, we need the combined effect of a high clean energy share, advanced technology level, and the development mode with low energy consumption. From the perspective of the world’s emission paths, except SSP5-3.4, the other four scenarios can achieve the peak and stable decline of CO_2_ emissions. In the scenarios of SSP3-3.4 and SSP4-3.4, the world can reach the peak as early as 2030.

For the single scenario:

(1) In the SSP1-3.4 scenario, except for Argentina, Brazil, India, Saudi Arabia, and South Africa, all other countries studied in this paper can achieve a peak in CO_2_ emissions by 2050. In this scenario, the CO_2_ emissions of China, India, Korea, Rep., Mexico, and Saudi Arabia are at a high level compared with other scenarios. The paths of the world also follow this pattern. This shows the challenge of achieving more stringent climate goals while adhering to the sustainable development path. Taking the world as an example, compared with the SSP1 baseline scenario, the CO_2_ emissions corresponding to the SSP1-3.4 scenario are lower. However, compared with other SSP-3.4 scenarios, SSP1-3.4 requires more resources to achieve sustainable development goals other than climate change, so its CO_2_ emissions will be higher.
(2) In the SSP2-3.4 scenario, most countries can reach the peak and achieve a stable decline in CO_2_ emissions. It is worth noting that the CO_2_ emissions of developed countries in the SSP2-3.4 scenario are generally higher than those in the SSP1-3.4 scenario which adheres to the sustainable development model. Due to the advanced technology level and development mode, developed countries can better balance the maintenance of the sustainable development path and the realization of climate goals. Therefore, compared with developing countries, climate goals will not encroach too much on the resources of other sustainable development goals.
(3) In the SSP3-3.4 scenario, the CO_2_ emission paths at both country and global levels are similar to those in the baseline scenario, and are generally at a relatively low level. This is obviously caused by insufficient development caused by vicious competition among countries. Although this scenario is beneficial to curb climate change, it is not conducive to achieving equitable resource allocation among countries to achieve common prosperity.
(4) For the SSP4-3.4 scenario, although it has the same connotation of inequality as SSP3-3.4, or even a higher degree of inequality, it is worth noting that this scenario does not exclude international cooperation. Similar to SSP4, it can achieve the goal of prosperity for some people. So, it can result in higher CO_2_ emissions than the lose–lose scenario of the SSP3-3.4. However, for the sustainable development scenario SSP1-3.4, where all people are treated equally, its emission levels will generally be lower.
(5) In the SSP5-3.4 scenario, similar to SSP5, CO_2_ emissions at both the country and global level are higher than in the other scenarios. Different from SSP5, in the SSP5-3.4 scenario, although countries still follow the development route of fossil energy consumption, the proportion of fossil energy will be lower than that of SSP5. At the same time, the consumption of primary energy is also lower, which can significantly reduce the level of energy intensity. For the above reasons, the emission levels of most countries show a convergence trend under the SSP5-3.4 scenario, that is, the peak time has been greatly advanced.

Overall, the five scenarios of SSP-3.4 strengthen the constraints on the energy consumption and fossil energy use through new climate policies, which will greatly increase the possibility of peaking CO_2_ emissions at both a country and global level. However, it is worth noting that the sustainable development path corresponding to SSP1-3.4 does not perform well compared with other emission paths corresponding to SSP-3.4, which conflicts with the conclusions drawn from the baseline scenarios. To achieve climate goals while maintaining other sustainable development goals, countries may need to pay more resources. It can be considered that climate goals have a crowding-out effect on other sustainable development goals. In view of this, how to maintain a balance between all-round development and climate goals should become an important topic of the climate summits in the future.

### 4.4. CO_2_ Emissions in 2050 Compared to 2019

**Figure 4:**
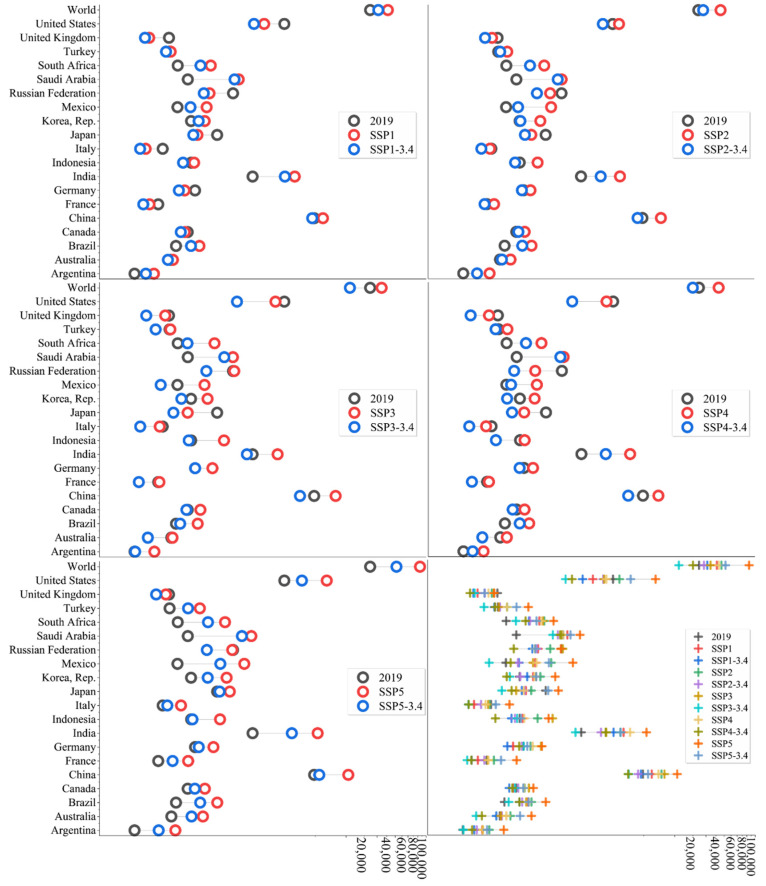
CO_2_ emissions in 2050 compared to 2019.

Figure 4 shows the comparison of CO_2_ emissions between 2050 and 2019 in the SSP baseline scenarios and SSP-3.4 scenarios. For the convenience of presentation, the horizontal axis of the graph uses a non-linear scale. From Figure 4, it can be seen that by 2050, the CO_2_ emissions corresponding to the SSP baseline scenarios are generally higher than those in the SSP-3.4 scenarios. This shows the importance of additional climate policies in achieving emission reduction goals. In addition, most countries will be able to achieve emission reductions in 2050 through at least one path.

To get a clearer picture of CO_2_ emissions in 2050, we report the rate of change of emissions in 2050 relative to 2019 in Table 4. We combine it with Figure 4 for analysis. It can be found that in the SSP baseline scenarios, only Canada, France, Germany, Italy, Japan, the Russian Federation, the United Kingdom, and the United States are likely to have lower CO_2_ emissions in 2050 than in 2019. All of the above countries can achieve emission reduction in the SSP1 scenario due to the higher level of development in history and the more advanced development model, as well as the impact of low energy consumption and low fossil energy growth in the future. It is worth noting that most of them can also achieve emission reduction in SSP3 and SSP4 scenarios. However, in order to achieve universal development and prosperity in the world, but also to achieve maximum emission reduction, it is clear that the SSP1 route with the connotation of sustainable development is worthier of praise. In terms of the world’s emission paths, the path corresponding to the SSP1 scenario is also the optimal choice.

In the SSP-3.4 scenarios, Australia, China, India, Indonesia, Korea, Rep., Mexico, and Turkey are the new countries that have a chance to achieve lower CO_2_ emissions in 2050 than in 2019. Among the above countries, Australia and Korea, Rep. are developed countries, but they are unable to achieve emission reduction in the baseline scenarios. The reason is that it is difficult for these two countries to achieve the significant optimization of energy structure in the baseline scenarios. China, Indonesia, and Turkey can achieve significant emission reductions in 2050 through at least three SSP-3.4 scenarios, but cannot achieve the maximum emission reduction through the SSP1-3.4 sustainable development path. Compared with the SSP baseline scenarios, this further verifies the crowding-out effect of climate targets on other sustainable development goals. It is worth noting that China, which currently accounts for the largest proportion of CO_2_ emissions in the world, can achieve its commitment to peak in 2030 in the SSP1-3.4, SSP3-3.4, and SSP5-3.4 scenarios, but cannot achieve emission reduction in 2050 in the SSP5-3.4 scenario. Obviously, the route corresponding to the SSP5-3.4 scenario cannot be chosen. In addition, India and Mexico can only achieve 2050 emission reductions under the SSP3-3.4 scenario, and the world can achieve emission reduction through the SSP3-3.4 and SSP4-3.4 scenarios. 

It should be noted that Argentina, Brazil, Saudi Arabia, and South Africa will have higher CO_2_ emissions in 2050 than in 2019 in both the SSP baseline and SSP-3.4 scenarios. This performance is mainly driven by a high energy consumption and high fossil energy consumption, although Argentina and Brazil are forecast to be unable to achieve lower CO_2_ emissions in 2050 than in 2019. However, combined with the previous analysis, it can be found that in the SSP-3.4 scenarios, the two countries can achieve the peak of CO_2_ emissions and maintain a steady decline in some paths. On the contrary, in Saudi Arabia and South Africa it will be difficult to maintain the downward trend of emissions.

## 5. Conclusions

Mitigating climate change requires long-term global efforts. The aim of this study is to simulate the possible paths of CO_2_ emissions in G20 countries and the world from 2020 to 2050. For this purpose, this paper first shows the historical CO_2_ emissions from 1990 to 2019, and then uses the STIRPAT model accompanied by PLS regression, from which the CO_2_ driving factors are analyzed. Then, through the method of scenario analysis, this paper explores the future emission paths at both a country and global level in the SSP baseline scenarios and SSP-3.4 scenarios, and then carries out the analysis. Finally, this paper compares the CO_2_ emissions in 2050 and 2019 in different scenarios.

Through the STIRPAT model, this paper finds that the population, economic development level, energy intensity, and the proportion of fossil energy representing the energy structure generally have a positive impact on CO_2_ emissions, while the coefficient of the urbanization rate shows heterogeneity among different countries. The urbanization rate of many developing countries has a positive effect on CO_2_ emissions, while in some developed countries with a high level of agricultural development, it has a negative effect. In addition, this paper finds that the coefficient of the quadratic term of economic development level is positive, which implies that there may be no inverted U-shaped relationship between per capita GDP and CO_2_ emissions.

In the part of scenario analysis, this paper first simulates the CO_2_ emission paths of all objects in 2020–2050 in the SSP baseline scenarios. It can be found that 13 countries can achieve the peak in at least one path, whereas six countries cannot reach the peak. Among them, the United Kingdom, France, Japan, Italy, the Russian Federation, and the United States can peak on multiple paths and keep emissions gradually decreasing. Some developing countries that achieved a peak in CO_2_ emissions between 1990 and 2019 cannot peak again in the predicted paths. This demonstrates the difficulty of balancing development and climate goals. As for the world, it cannot reach the peak through any path. In all scenarios, the vicious competition route corresponding to SSP3 can achieve the lowest emission level, which depends on the low level of economic development. This is obviously not conducive to the prosperity of the world. Generally speaking, on the premise of keeping the existing climate policies unchanged, the sustainable development path corresponding to SSP1 is the worthiest of praise. At the same time, in any case, the world should avoid the path represented by high fossil energy consumption and high energy intensity corresponding to SSP5.

With SSP-3.4 scenarios, it can be found that the reduction of the energy intensity and the proportion of fossil energy can greatly advance the peak time of CO_2_ emissions. In this setting, only Saudi Arabia cannot reach the peak. In terms of path selection, there are different results from the SSP baseline scenarios simulation. Although SSP1-3.4, which represents the sustainable development path, generally represents a relatively low emission level in developed countries, it cannot correspond to the lowest emission level. Developing countries perform even worse. The performance at the global level is also bad. This suggests that achieving climate goals may have a crowding-out effect on achieving other sustainable development goals. In terms of the future development route setting, there is no doubt that the Sustainable Development Goals (SDGs) adopted by the 193 members of the United Nations are the worthiest of praise. This requires all countries in the world to establish a balance between achieving climate goals and achieving other sustainable development goals. In order to solve the difficulties faced by emerging economies and developing countries in this regard, more help from developed countries may be needed. 

Finally, this paper looks forward to the CO_2_ emissions at both a country and global level in 2050, and compares them with the emissions in 2019. We find that the SSP-3.4 scenario with more stringent climate policy constraints is more likely to achieve emission reductions than the SSP baseline scenarios. In the SSP-3.4 scenarios, except for Argentina, Brazil, Saudi Arabia, and South Africa, other countries can achieve substantial emission reductions through at least one path in 2050. For the world, emission reduction can also be achieved in 2050 in the scenarios of SSP3-3.4 and SSP4-3.4. In all SSP-3.4 scenarios, SSP1-3.4 cannot achieve the maximum emission reduction. This confirms once again the crowding-out effect of climate goals on other sustainable development goals. Its impact on developing countries is greater than on developed countries. Combined with the scenario analysis above, it is found that it will be difficult for Saudi Arabia and South Africa to achieve peak and emission reduction by 2050. To change this situation, these two countries need to optimize their energy structure and reduce the energy intensity in the future.

In summary, curbing climate change is the common responsibility of the whole world. No country can be immune from the impact of extreme climate. In order to effectively control CO_2_ emissions, we believe that countries in the world should extensively establish a cooperative framework for sustainable development. Through this framework, emerging economies and developing countries can have sufficient ways to obtain advanced technology or other support, so as to reduce the energy intensity and the proportion of fossil energy, and reduce the crowding-out effect of climate goals. In addition to participating in the worldwide cooperation framework, countries should also improve their domestic laws to limit high energy consumption technologies in the production process. Countries also need to make rational planning for the future development mode to gradually reduce the use of fossil energy.

## 6. Limitation

Since the main purpose of this paper is to explore the path of CO_2_ emissions, only six main influencing factors are included in the model to investigate the relationship between CO_2_ emissions and its influencing factors, without individualized customization. In addition, the IIASA database only provides quantified values of the main influencing factors. While in the qualitative scenario setting of SSP, there are still more quantification possibilities of influencing factors of CO_2_ emissions. Therefore, in future research, researchers can add more interesting variables into the model according to the needs of the research topic, when historical data are available and SSP scenarios have their corresponding descriptions. In addition, for the setting of the SSP extension scenario, this paper only combines RCP3.4. In future studies, researchers can combine more different RCP scenarios with SSP according to their research purposes, such as RCP1.9, with the most severe limit on the irradiation forcing level, or RCP4.5, with the less stringent limit.

## Figures and Tables

**Table 1:** Interpretation of variables.

Variable	Meaning	Description
CE	CO_2_ emissions	Annual CO_2_ emissions, Mt
P	Population size	Year-end population
A	Economic development level	Per capita GDP, at constant 2015 purchasing power parity (PPP), USD
EI	Energy intensity	Total primary energy consumption/GDP, tce/10^5^
UR	Urbanization rate	Urban population/Total population, %
FS	Proportion of fossil energy consumption	Fossil energy consumption/Total primary energy consumption, %

**Table 2:** Changes of CO_2_ emission during 1990–2019 (Mt).

Country/Region	1990	2000	2010	2019	Proportion (%)	Growth Rate (%)
Argentina	105.452	132.610	168.807	175.818	0.512	1.778
Australia	274.888	355.033	396.535	400.973	1.167	1.310
Brazil	198.637	306.378	403.094	444.906	1.295	2.820
Canada	449.773	537.970	550.117	577.997	1.682	0.869
China	2323.833	3360.874	8145.828	9810.456	28.555	5.092
France	367.241	381.500	360.362	298.951	0.870	−0.707
Germany	1007.606	854.428	783.163	681.483	1.984	−1.339
India	613.130	961.256	1652.135	2471.946	7.195	4.925
Indonesia	145.051	278.152	446.455	624.547	1.818	5.163
Italy	403.781	434.379	397.116	330.276	0.961	−0.691
Japan	1086.992	1233.184	1197.903	1117.673	3.253	0.096
Korea, Rep.	235.438	428.597	578.895	623.159	1.814	3.413
Mexico	269.854	363.004	454.822	459.759	1.338	1.854
Russian Federation	2233.921	1452.763	1526.638	1595.686	4.644	−1.153
Saudi Arabia	217.149	279.310	471.961	579.622	1.687	3.443
South Africa	324.870	371.650	474.864	462.448	1.346	1.225
Turkey	136.240	205.693	276.300	385.453	1.122	3.651
United Kingdom	600.322	569.793	529.970	380.175	1.107	−1.563
United States	4978.861	5745.765	5494.979	5029.389	14.639	0.035
World	21,548.909	23,847.931	31,291.429	34,356.612	100.000	1.622

**Table 3:** Results of PLS analysis based on STIRPAT model.

Country/Region	Cons	lnP	lnA	(lnA)^2^	lnEI	lnUR	lnFS
Argentina	−29.325	0.484	0.399	0.021	0.655	2.609	1.466
Australia	−1.396	0.279	0.345	0.016	−0.108	−3.237	2.566
Brazil	−21.179	0.482	0.548	0.031	1.071	0.771	0.929
Canada	−17.591	0.060	0.344	0.016	0.607	2.642	0.877
China	−40.416	1.415	0.327	0.021	1.023	0.930	1.700
France	−37.518	1.807	0.438	0.020	1.043	−0.777	1.160
Germany	−2.169	−0.175	0.578	0.028	1.175	−0.812	0.715
India	−24.557	0.582	0.380	0.029	1.075	1.753	1.189
Indonesia	−57.171	2.597	0.436	0.037	1.149	−0.884	1.490
Italy	−30.039	1.550	0.457	0.023	0.785	−1.569	1.280
Japan	−67.089	3.466	0.347	0.017	0.608	−0.366	0.906
Korea, Rep.	−42.889	1.198	0.186	0.009	0.138	3.445	2.139
Mexico	−36.284	0.435	0.512	0.028	0.456	1.547	4.217
Russian Federation	−34.814	2.320	0.558	0.027	0.975	−4.444	1.382
Saudi Arabia	−31.784	0.434	0.733	0.037	0.692	3.821	0.000
South Africa	−27.411	0.648	0.490	0.028	1.028	0.879	1.718
Turkey	−17.434	0.430	0.495	0.028	0.867	0.579	0.727
United Kingdom	12.985	−0.620	0.108	0.005	0.093	−1.304	1.823
United States	−51.993	2.663	0.507	0.019	1.145	−3.515	2.867
World	−17.762	0.249	0.472	0.027	0.921	1.034	1.961

**Table 4:** Growth rate of CO_2_ emissions in 2050 compared to 2019.

Country	SSP1	SSP2	SSP3	SSP4	SSP5	SSP1-3.4	SSP2-3.4	SSP3-3.4	SSP4-3.4	SSP5-3.4
Argentina	54.50	80.53	55.99	57.01	149.53	28.78	36.33	1.36	22.94	71.79
Australia	3.61	26.76	2.53	16.00	102.87	−7.43	4.43	−40.94	−33.05	57.22
Brazil	69.09	82.95	62.53	72.48	151.91	39.84	48.59	9.84	39.50	71.85
Canada	−6.21	20.76	32.94	19.63	46.87	−14.31	4.90	−2.80	−8.33	17.30
China	21.79	51.41	61.69	41.35	114.84	−4.30	−10.32	−27.17	−28.13	12.15
France	−17.80	17.55	2.62	4.15	95.43	−28.46	−5.10	−35.26	−28.88	37.91
Germany	−20.84	16.73	47.61	22.62	50.96	−30.39	−3.81	0.26	−9.15	8.71
India	157.05	139.77	75.13	197.83	329.27	106.01	55.56	−12.00	72.47	140.79
Indonesia	6.90	49.39	108.72	10.95	90.94	−16.60	−9.89	−5.31	−41.74	2.97
Italy	−31.71	−3.03	−6.37	−11.61	50.76	−39.67	−20.12	−39.55	−39.34	10.95
Japan	−35.69	−27.53	−48.31	−38.64	32.81	−41.33	−37.20	−62.46	−53.29	5.71
Korea, Rep.	35.77	59.20	44.73	39.10	121.78	18.65	1.77	−19.52	−24.97	45.20
Mexico	92.19	174.92	83.29	98.30	346.42	33.93	30.90	−31.30	11.16	161.13
Russian Federation	−40.96	−22.46	2.76	−44.99	−1.84	−48.05	−42.42	−45.01	−65.66	−44.38
Saudi Arabia	212.52	176.04	174.62	186.38	314.74	184.73	152.93	126.19	166.11	235.75
South Africa	109.57	134.10	127.97	118.33	188.18	66.55	70.10	24.43	54.33	96.92
Turkey	2.47	22.49	1.78	22.18	96.87	−7.68	4.50	−26.82	−6.26	51.19
United Kingdom	−35.80	−11.75	−8.05	−18.00	−6.74	−41.54	−24.60	−39.87	−45.65	−25.01
United States	−36.31	15.28	−17.99	−13.77	158.65	−49.29	−19.82	−65.34	−60.01	48.34
World	48.76	64.89	29.37	55.66	205.42	20.06	11.50	−36.37	−12.77	80.30

## Data Availability

The datasets used in this study are available from the corresponding author on reasonable request.

## Supplementary Materials

The following supporting information can be downloaded at: https://www.mdpi.com/article/10.3390/ijerph191711076/s1, Figure S1: Results of t1/t2 test; Figure S2: Results of t1/u1 test; Figure S3: The variable importance in projection (VIP) of each factor; Table S1: VIF values; Table S2: The summary of PLS regression; Tables S3.1–20: Scenario setup for each country and the world.
