# Supplementary material for: Peaking Global and G20 Countries’ CO2 Emissions under the Shared Socio-Economic Pathways
Source: Int J Environ Res Public Health. 2022 Sep 4;19(17):11076. doi: 10.3390/ijerph191711076 (PMC9518017; doi:10.3390/ijerph191711076)
Supplement: Supplementary file 1 [file ijerph-19-11076-s001.zip › ijerph-1861133-supplementary.pdf]

Peaking global and G20 Countries’ CO2 emissions under the shared socio-economic pathways

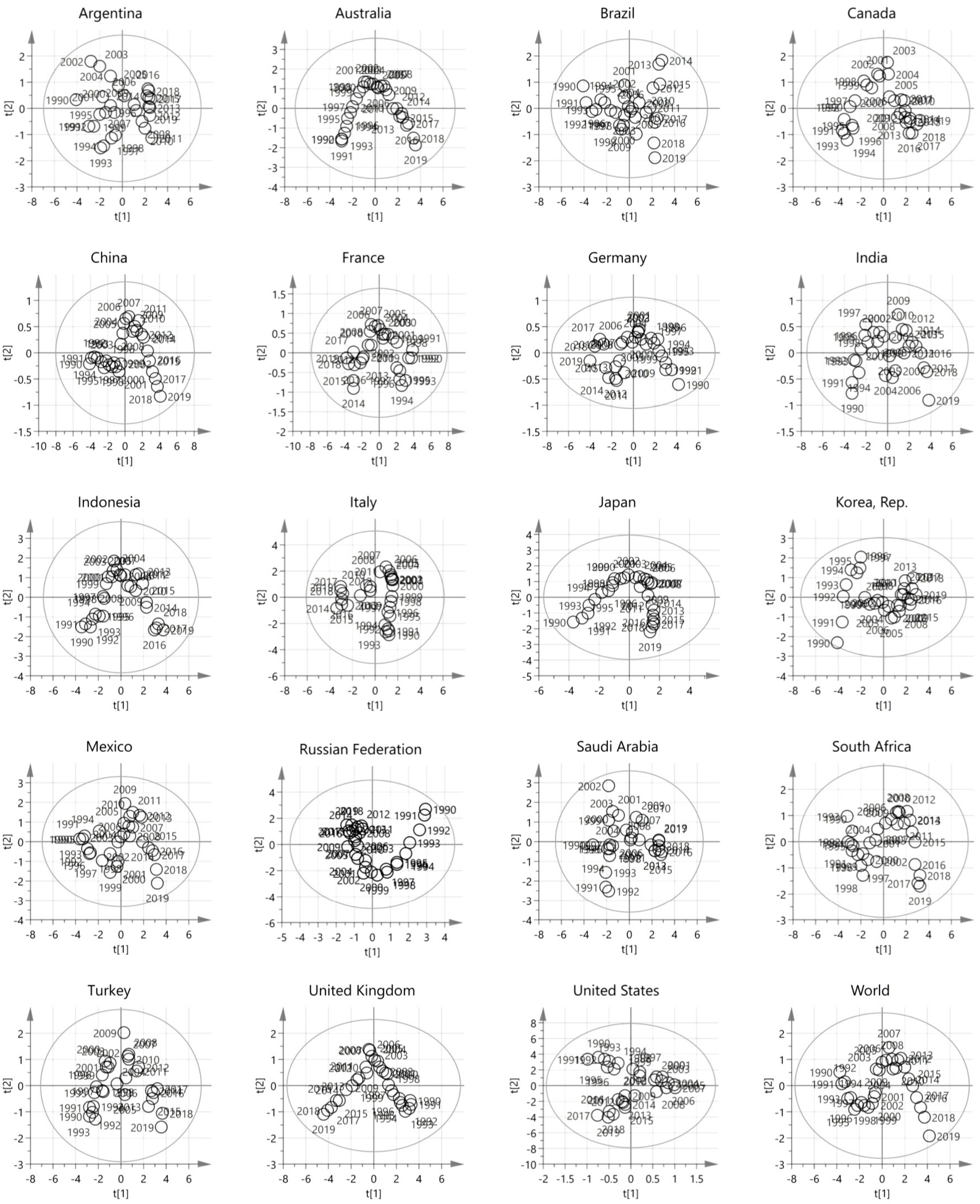

Figure S1. Results of t1/t2 test

Note: t1 and t2 are the latent variables extracted from independent variables.

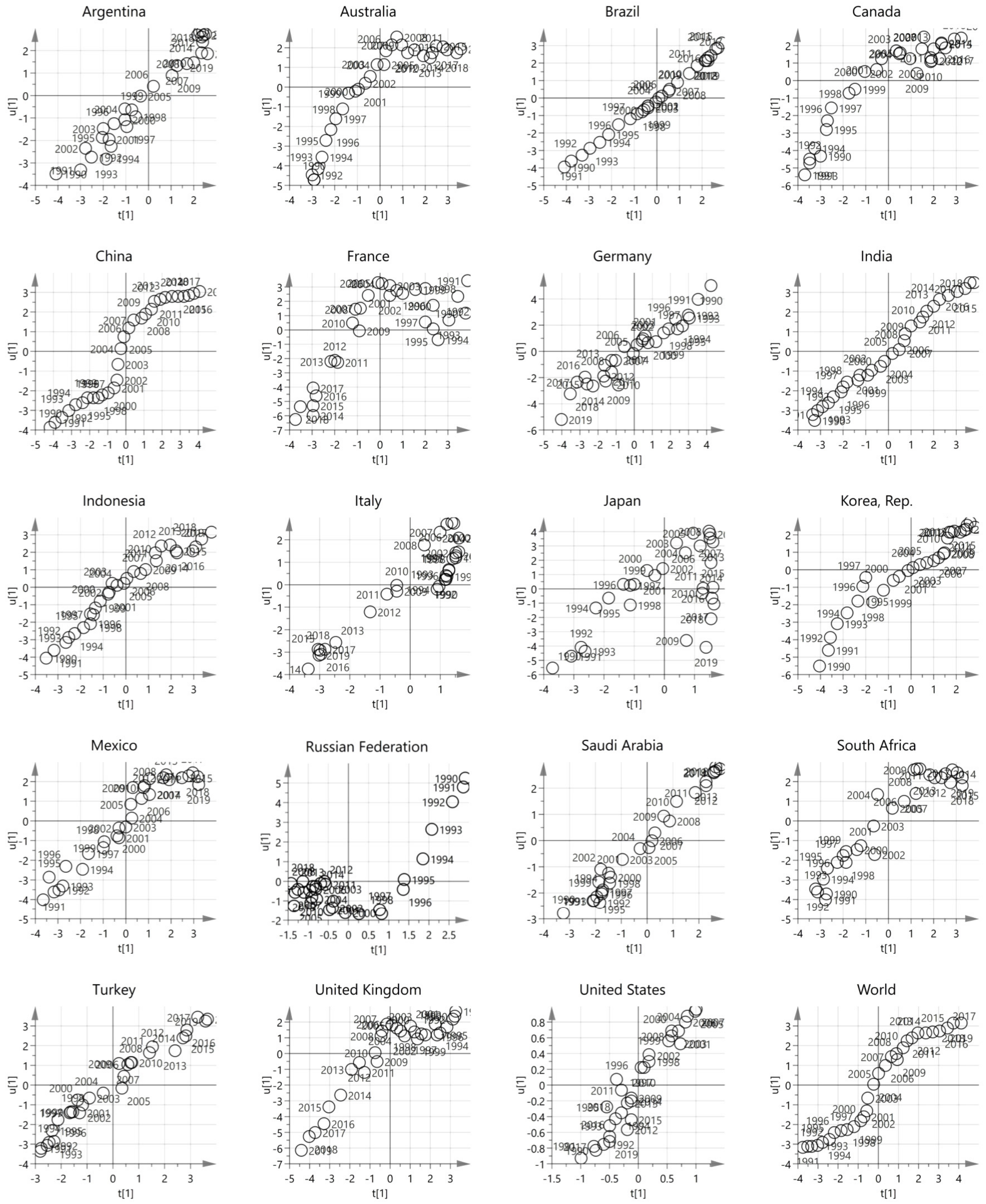

Figure S2. Results of t1/u1 test

Note: t1, u1 are the latent variable extracted from independent variables and dependent variables, respectively.

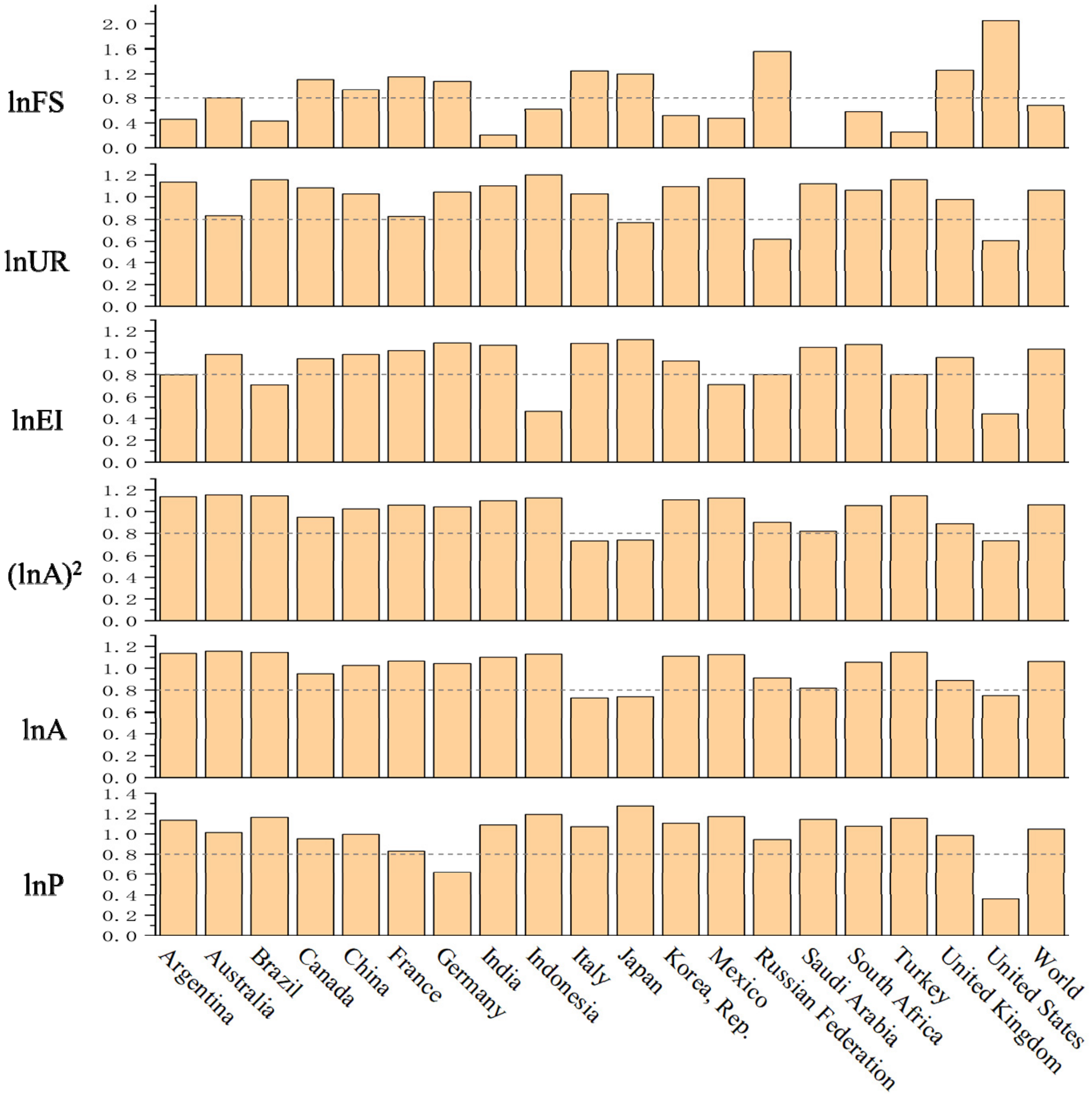

Figure S3. The variable importance in projection (VIP) of each factor

Table S1. VIF values

**Countries**	**lnP**	**lnA**	**$(lnA)^2$**	**lnEI**	**lnUR**	**lnFS**
**Argentina**	307.96	21772.04	22160.43	6.04	389.3	2.3
**Australia**	170.65	862338.5	868676	129.29	32.1	10.23
**Brazil**	254.28	141290.73	139234.2	3.29	289.3	1.7
**Canada**	103.33	347206.09	350246.75	58.69	88.75	3.77
**China**	2366.98	34610.18	37585.93	87.31	3153.93	52.26
**France**	145.26	274398.88	277039.78	82.13	272.48	6.27
**Germany**	2.51	243149.56	240169.48	76.32	55.7	47.38
**India**	894.56	35768.21	36477.19	28.38	1616.01	2.14
**Indonesia**	701.98	33119.15	34169.36	10.71	544.41	4.28
**Italy**	13.22	219716.47	220148.52	31.97	19.49	25.75
**Japan**	24.21	833037.44	831929.38	35.51	28.19	4.36
**Korea, Rep.**	179.12	85188.42	84422.49	50.9	113.17	3.61
**Mexico**	3500.4	76012.21	76892.51	3.6	3368.81	1.55
**Russian Federation**	29.29	78359.81	88130.07	402.76	32.16	43.74
**Saudi Arabia**	219.35	102029.66	102603.31	21.39	123.02	2.18
**South Africa**	554.11	93621.06	95169.93	21.73	860.15	2.6
**Turkey**	538.48	21847.5	21710.24	4.59	639.79	1.95
**United Kingdom**	1082.52	144413.91	145076.7	260.73	853.9	9.24
**United States**	792.87	239929.14	232390.08	295.42	627.58	26.33
**World**	3807.93	463714.84	460090	161.64	3400.25	18.46

Table S2. The summary of PLS regression

Country	lnP	lnA	$(\ln A)^2$	lnEI	lnUR	lnFS	R^2X (cum)	R^2Y (cum)	Q^2 (cum)
Argentina	0.231*** (0.014)	0.322*** (0.026)	0.323*** (0.025)	0.172*** (0.029)	0.219*** (0.035)	0.157*** (0.015)	1	0.996	0.995
Australia	0.248*** (0.032)	0.413*** (0.037)	0.41*** (0.037)	-0.111 (0.102)	-0.159*** (0.041)	0.265*** (0.049)	0.997	0.987	0.986
Brazil	0.187*** (0.01)	0.289*** (0.01)	0.292*** (0.01)	0.155*** (0.01)	0.134*** (0.01)	0.106*** (0.01)	0.999	0.999	0.999
Canada	0.059(0.13)	0.53*** (0.114)	0.521*** (0.115)	0.972*** (0.207)	0.594*** (0.139)	0.241*** (0.028)	0.998	0.989	0.982
China	0.165*** (0.03)	0.474*** (0.017)	0.499*** (0.022)	0.548*** (0.019)	0.461*** (0.011)	0.109*** (0.014)	0.999	0.999	0.999
France	1.002*** (0.234)	0.495*** (0.018)	0.467*** (0.023)	1.853*** (0.063)	-0.24 (0.21)	0.669*** (0.03)	1	0.998	0.996
Germany	-0.022* (0.019)	0.747*** (0.071)	0.762*** (0.078)	2.009*** (0.135)	-0.177* (0.092)	0.29* (0.161)	1	0.994	0.987
India	0.18*** (0.031)	0.357*** (0.016)	0.372*** (0.016)	0.277*** (0.02)	0.362*** (0.018)	0.02*** (0.006)	1	1	1
Indonesia	0.737*** (0.069)	0.286*** (0.027)	0.379*** (0.027)	0.311*** (0.015)	-0.376*** (0.062)	0.026*** (0.008)	1	0.999	0.999
Italy	0.34*** (0.114)	0.243*** (0.026)	0.248*** (0.025)	0.52*** (0.154)	-0.234 (0.144)	0.529*** (0.142)	0.988	0.989	0.987
Japan	0.678*** (0.123)	0.453*** (0.141)	0.448*** (0.142)	1.295*** (0.202)	-0.496*** (0.161)	0.872*** (0.112)	0.997	0.941	0.884
Korea, Rep.	0.243*** (0.015)	0.247*** (0.013)	0.24*** (0.013)	0.056 (0.041)	0.361*** (0.019)	0.13*** (0.018)	0.997	0.993	0.989
Mexico	0.278*** (0.027)	0.223*** (0.017)	0.222*** (0.017)	0.115** (0.05)	0.276*** (0.027)	0.182*** (0.037)	0.913	0.968	0.963
Russian Federation	0.303*** (0.079)	1.344***(0.063)	1.138***(0.037)	2.057*** (0.135)	-0.188*** (0.055)	0.158*** (0.053)	1	0.997	0.995
Saudi Arabia	0.284*** (0.01)	0.13*** (0.01)	0.131*** (0.01)	0.29*** (0.017)	0.278*** (0.008)	0	0.988	0.993	0.992
South Africa	0.61*** (0.038)	0.422*** (0.015)	0.416*** (0.016)	0.919*** (0.024)	0.477*** (0.031)	0.076*** (0.012)	1	0.999	0.999
Turkey	0.166*** (0.027)	0.397*** (0.028)	0.403*** (0.032)	0.133*** (0.017)	0.129*** (0.045)	0.057*** (0.017)	1	0.998	0.997
United Kingdom	-0.23*** (0.021)	0.11*** (0.022)	0.107*** (0.022)	0.161*** (0.012)	-0.224*** (0.022)	0.597*** (0.033)	0.979	0.981	0.979
United States	3.935*** (0.708)	1.213*** (0.171)	1.005*** (0.186)	3.676*** (0.43)	-1.618*** (0.539)	0.971*** (0.099)	1	0.986	0.877
World	0.16** (0.058)	0.428*** (0.038)	0.451*** (0.038)	0.49*** (0.044)	0.477*** (0.048)	0.092*** (0.006)	0.999	1	1

Note: the value in () is the standard deviation, ***p<0.01, ***p<0.05, and ***p<0.1.

R2X(cum): Cumulative X variance

R2Y(cum): Cumulative Y variance (R-square)

Q2(cum): Cumulative Q square (the cross-validity value)

Table S3.1. Scenario setup for Argentina

Period		2019-2020	2021-2025	2026-2030	2031-2035	2036-2040	2041-2045	2046-2050
P	SSP1	0.556	0.420	0.296	0.216	0.120	0.010	-0.096
	SSP2	0.715	0.621	0.536	0.457	0.388	0.294	0.213
	SSP3	0.921	0.906	0.872	0.803	0.755	0.720	0.694
	SSP4	0.616	0.476	0.335	0.244	0.140	0.024	-0.093
	SSP5	0.520	0.366	0.242	0.161	0.065	-0.044	-0.151
GDP	SSP1	3.768	3.735	3.601	3.407	2.962	2.462	2.053
	SSP2	3.824	3.454	3.112	2.802	2.466	2.172	2.011
	SSP3	3.919	3.126	2.465	1.944	1.594	1.320	1.240
	SSP4	3.703	3.184	2.848	2.485	2.060	1.677	1.503
	SSP5	3.777	4.084	4.334	4.229	3.615	3.029	2.584
UR	SSP1	0.170	0.150	0.132	0.116	0.102	0.089	0.079
	SSP2	0.170	0.150	0.132	0.116	0.102	0.089	0.079
	SSP3	0.170	0.150	0.132	0.116	0.102	0.089	0.079
	SSP4	0.170	0.150	0.132	0.116	0.102	0.089	0.079
	SSP5	0.170	0.150	0.132	0.116	0.102	0.089	0.079
Primary energy consumption	SSP1	2.473	1.654	1.654	1.710	1.710	1.298	1.298
	SSP2	2.139	1.897	1.897	1.820	1.820	1.249	1.249
	SSP3	2.532	2.154	2.154	1.493	1.493	1.231	1.231
	SSP4	2.967	2.301	2.301	1.477	1.477	0.935	0.935
	SSP5	3.098	2.345	2.345	1.895	1.895	1.722	1.722
	SSP1-3.4	2.476	1.421	1.421	1.161	1.161	0.928	0.928
	SSP2-3.4	1.933	1.629	1.629	1.704	1.704	1.188	1.188
	SSP3-3.4	2.541	2.123	2.123	-0.067	-0.067	0.997	0.997
	SSP4-3.4	2.491	1.953	1.953	1.209	1.209	0.712	0.712
	SSP5-3.4	3.098	1.911	1.911	0.713	0.713	1.168	1.168
Fossil energy consumption	SSP1	3.006	1.745	1.745	1.149	1.149	0.970	0.970
	SSP2	2.111	2.293	2.293	2.130	2.130	1.088	1.088
	SSP3	2.636	2.289	2.289	1.515	1.515	1.134	1.134
	SSP4	3.096	2.229	2.229	1.320	1.320	0.769	0.769
	SSP5	3.242	3.391	3.391	2.639	2.514	1.688	1.688
	SSP1-3.4	3.048	1.431	1.431	0.330	0.330	0.214	0.214
	SSP2-3.4	1.725	1.276	1.276	1.314	1.314	0.755	0.755
	SSP3-3.4	2.610	2.170	2.170	-1.046	-1.046	-0.138	-0.138
	SSP4-3.4	2.305	1.533	1.533	0.719	0.719	-0.029	-0.029
	SSP5-3.4	3.224	2.758	2.758	1.022	1.022	0.088	0.088

Table S3.2. Scenario setup for Australia

Period		2019-2020	2021-2025	2026-2030	2031-2035	2036-2040	2041-2045	2046-2050
P	SSP1	1.509	1.401	1.259	1.153	1.082	1.012	0.934
	SSP2	1.486	1.373	1.222	1.102	1.023	0.960	0.893
	SSP3	1.055	0.753	0.557	0.435	0.343	0.253	0.148
	SSP4	1.395	1.251	1.070	0.955	0.863	0.775	0.679
	SSP5	1.903	1.948	1.812	1.671	1.594	1.547	1.495
GDP	SSP1	3.419	3.040	2.874	2.786	2.618	2.450	2.158
	SSP2	3.358	2.806	2.404	2.158	2.049	1.982	1.801
	SSP3	2.918	2.178	1.625	1.262	1.025	0.852	0.582
	SSP4	3.303	2.958	2.795	2.703	2.520	2.355	2.080
	SSP5	3.833	3.774	3.893	3.902	3.719	3.548	3.266
UR	SSP1	0.176	0.162	0.149	0.137	0.126	0.115	0.106
	SSP2	0.176	0.162	0.149	0.137	0.126	0.115	0.106
	SSP3	0.176	0.162	0.149	0.137	0.126	0.115	0.106
	SSP4	0.176	0.162	0.149	0.137	0.126	0.115	0.106
	SSP5	0.176	0.162	0.149	0.137	0.126	0.115	0.106
Primary energy consumption	SSP1	-0.097	-0.319	-0.319	-0.049	-0.049	-0.227	-0.227
	SSP2	0.562	0.436	0.436	0.479	0.479	0.489	0.489
	SSP3	1.130	0.633	0.633	0.377	0.377	0.256	0.256
	SSP4	1.007	0.929	0.929	0.535	0.535	0.200	0.200
	SSP5	0.706	1.592	1.592	1.592	1.592	1.493	1.493
	SSP1-3.4	-0.121	-0.466	-0.466	-0.461	-0.461	-0.469	-0.469
	SSP2-3.4	0.434	0.110	0.110	0.078	0.078	0.069	0.069
	SSP3-3.4	0.726	-0.874	-0.874	-0.250	-0.250	0.498	0.498
	SSP4-3.4	0.725	0.467	0.467	0.063	0.063	-0.059	-0.059
	SSP5-3.4	0.565	1.007	1.007	0.574	0.574	0.953	0.953
Fossil energy consumption	SSP1	-0.322	-0.741	-0.741	-0.375	-0.375	-0.711	-0.711
	SSP2	0.239	0.389	0.389	0.535	0.535	0.529	0.529
	SSP3	1.149	0.578	0.578	0.293	0.293	0.132	0.132
	SSP4	0.731	0.683	0.683	0.276	0.276	-0.013	-0.013
	SSP5	0.604	2.074	2.074	1.935	1.935	1.585	1.577
	SSP1-3.4	-0.324	-0.884	-0.884	-0.958	-0.958	-1.253	-1.253
	SSP2-3.4	0.060	-0.030	-0.030	-0.093	-0.093	-0.371	-0.371
	SSP3-3.4	0.621	-1.584	-1.584	-1.563	-1.563	0.061	0.061
	SSP4-3.4	0.249	-0.308	-0.308	-0.951	-0.951	-1.149	-1.149
	SSP5-3.4	0.424	1.334	1.334	0.661	0.661	0.357	0.357

Table S3.3. Scenario setup for Brazil

Period		2019-2020	2021-2025	2026-2030	2031-2035	2036-2040	2041-2045	2046-2050
P	SSP1	0.583	0.437	0.279	0.160	0.037	-0.079	-0.206
	SSP2	0.737	0.626	0.492	0.367	0.254	0.147	0.029
	SSP3	0.892	0.827	0.730	0.605	0.512	0.440	0.366
	SSP4	0.636	0.477	0.293	0.151	0.009	-0.131	-0.280
	SSP5	0.566	0.412	0.254	0.135	0.014	-0.101	-0.229
GDP	SSP1	3.955	3.694	3.573	3.501	3.230	2.843	2.420
	SSP2	4.001	3.283	2.682	2.274	2.099	1.913	1.722
	SSP3	4.052	2.945	1.996	1.301	1.021	0.769	0.545
	SSP4	3.880	3.097	2.590	2.201	1.981	1.734	1.496
	SSP5	3.992	4.109	4.356	4.422	4.022	3.544	3.055
UR	SSP1	0.223	0.184	0.154	0.131	0.112	0.097	0.085
	SSP2	0.223	0.184	0.154	0.131	0.112	0.097	0.085
	SSP3	0.223	0.184	0.154	0.131	0.112	0.097	0.085
	SSP4	0.223	0.184	0.154	0.131	0.112	0.097	0.085
	SSP5	0.223	0.184	0.154	0.131	0.112	0.097	0.085
Primary energy consumption	SSP1	2.473	1.654	1.654	1.710	1.710	1.298	1.298
	SSP2	2.139	1.897	1.897	1.820	1.820	1.249	1.249
	SSP3	2.532	2.154	2.154	1.493	1.493	1.231	1.231
	SSP4	2.967	2.301	2.301	1.477	1.477	0.935	0.935
	SSP5	3.098	2.345	2.345	1.895	1.895	1.722	1.722
	SSP1-3.4	2.476	1.421	1.421	1.161	1.161	0.928	0.928
	SSP2-3.4	1.933	1.629	1.629	1.704	1.704	1.188	1.188
	SSP3-3.4	2.541	2.123	2.123	-0.067	-0.067	0.997	0.997
	SSP4-3.4	2.491	1.953	1.953	1.209	1.209	0.712	0.712
	SSP5-3.4	3.098	1.911	1.911	0.713	0.713	1.168	1.168
Fossil energy consumption	SSP1	3.006	1.745	1.745	1.149	1.149	0.970	0.970
	SSP2	2.111	2.293	2.293	2.130	2.130	1.088	1.088
	SSP3	2.636	2.289	2.289	1.515	1.515	1.134	1.134
	SSP4	3.096	2.229	2.229	1.320	1.320	0.769	0.769
	SSP5	3.242	3.391	3.391	2.639	2.639	1.688	1.688
	SSP1-3.4	3.048	1.431	1.431	0.330	0.330	0.214	0.214
	SSP2-3.4	1.725	1.276	1.276	1.314	1.314	0.755	0.755
	SSP3-3.4	2.610	2.170	2.170	-1.046	-1.046	-0.138	-0.138
	SSP4-3.4	2.305	1.533	1.533	0.719	0.719	-0.029	-0.029
	SSP5-3.4	3.224	2.758	2.758	1.022	1.022	0.088	0.088

Table S3.4. Scenario setup for Canada

Period		2019-2020	2021-2025	2026-2030	2031-2035	2036-2040	2041-2045	2046-2050
P	SSP1	1.088	1.036	0.944	0.860	0.808	0.772	0.728
	SSP2	1.041	0.975	0.864	0.765	0.710	0.677	0.650
	SSP3	0.649	0.397	0.218	0.089	-0.009	-0.093	-0.176
	SSP4	0.948	0.843	0.698	0.599	0.520	0.449	0.376
	SSP5	1.432	1.530	1.466	1.368	1.330	1.326	1.319
GDP	SSP1	2.556	1.903	1.779	1.936	1.958	1.888	1.747
	SSP2	2.501	2.036	1.903	1.914	1.872	1.742	1.578
	SSP3	2.119	1.482	1.374	1.535	1.604	1.439	1.132
	SSP4	2.418	1.943	1.955	2.111	2.010	1.802	1.570
	SSP5	2.888	2.773	3.187	3.630	3.641	3.458	3.169
UR	SSP1	0.377	0.339	0.304	0.274	0.246	0.222	0.200
	SSP2	0.377	0.339	0.304	0.274	0.246	0.222	0.200
	SSP3	0.377	0.339	0.304	0.274	0.246	0.222	0.200
	SSP4	0.377	0.339	0.304	0.274	0.246	0.222	0.200
	SSP5	0.377	0.339	0.304	0.274	0.246	0.222	0.200
Primary energy consumption	SSP1	-0.097	-0.319	-0.319	-0.049	-0.049	-0.227	-0.227
	SSP2	0.562	0.436	0.436	0.479	0.479	0.489	0.489
	SSP3	1.130	0.633	0.633	0.377	0.377	0.256	0.256
	SSP4	1.007	0.929	0.929	0.535	0.535	0.200	0.200
	SSP5	0.706	1.592	1.592	1.592	1.592	1.493	1.493
	SSP1-3.4	-0.121	-0.466	-0.466	-0.461	-0.461	-0.469	-0.469
	SSP2-3.4	0.434	0.110	0.110	0.078	0.078	0.069	0.069
	SSP3-3.4	0.726	-0.874	-0.874	-0.250	-0.250	0.498	0.498
	SSP4-3.4	0.725	0.467	0.467	0.063	0.063	-0.059	-0.059
	SSP5-3.4	0.565	1.007	1.007	0.574	0.574	0.953	0.953
Fossil energy consumption	SSP1	-0.322	-0.741	-0.741	-0.375	-0.375	-0.711	-0.711
	SSP2	0.239	0.389	0.389	0.535	0.535	0.529	0.529
	SSP3	1.149	0.578	0.578	0.293	0.293	0.132	0.132
	SSP4	0.731	0.683	0.683	0.276	0.276	-0.013	-0.013
	SSP5	0.604	2.074	2.074	1.935	1.935	1.585	1.585
	SSP1-3.4	-0.324	-0.884	-0.884	-0.958	-0.958	-1.253	-1.253
	SSP2-3.4	0.060	-0.030	-0.030	-0.093	-0.093	-0.371	-0.371
	SSP3-3.4	0.621	-1.584	-1.584	-1.563	-1.563	0.061	0.061
	SSP4-3.4	0.249	-0.308	-0.308	-0.951	-0.951	-1.149	-1.149
	SSP5-3.4	0.424	1.334	1.334	0.661	0.661	0.357	0.357

Table S3.5. Scenario setup for China

Period		2019-2020	2021-2025	2026-2030	2031-2035	2036-2040	2041-2045	2046-2050
P	SSP1	0.156	-0.005	-0.166	-0.304	-0.453	-0.596	-0.733
	SSP2	0.231	0.086	-0.065	-0.224	-0.379	-0.520	-0.652
	SSP3	0.292	0.166	0.024	-0.144	-0.294	-0.411	-0.500
	SSP4	0.134	-0.046	-0.230	-0.398	-0.573	-0.743	-0.911
	SSP5	0.155	-0.006	-0.166	-0.304	-0.452	-0.595	-0.730
GDP	SSP1	8.206	7.037	5.811	4.528	3.318	2.405	1.557
	SSP2	7.978	5.982	4.271	2.987	2.310	1.827	1.314
	SSP3	7.881	5.384	3.337	1.847	1.228	0.814	0.370
	SSP4	7.914	5.975	4.397	3.095	2.320	1.737	1.132
	SSP5	8.443	7.977	6.945	5.446	3.904	2.832	1.923
UR	SSP1	2.018	1.651	1.353	1.113	0.921	0.767	0.643
	SSP2	1.400	1.180	0.999	0.850	0.727	0.625	0.541
	SSP3	0.768	0.644	0.542	0.458	0.388	0.331	0.283
	SSP4	2.018	1.651	1.353	1.113	0.921	0.767	0.643
	SSP5	2.018	1.651	1.353	1.113	0.921	0.767	0.643
Primary energy consumption	SSP1	1.992	1.904	1.904	1.445	1.445	0.784	0.784
	SSP2	2.195	1.915	1.915	1.307	1.307	1.302	1.302
	SSP3	3.284	2.497	2.497	1.437	1.437	1.030	1.030
	SSP4	3.546	2.448	2.448	1.211	1.211	0.515	0.515
	SSP5	3.294	3.194	3.194	2.373	2.373	1.756	1.756
	SSP1-3.4	1.989	1.649	1.649	0.757	0.757	0.247	0.247
	SSP2-3.4	2.083	1.337	1.337	0.627	0.627	0.708	0.708
	SSP3-3.4	3.254	2.188	2.188	-1.524	-1.524	0.671	0.671
	SSP4-3.4	3.452	1.409	1.409	0.653	0.653	0.063	0.063
	SSP5-3.4	3.214	2.212	2.212	0.868	0.868	0.758	0.758
Fossil energy consumption	SSP1	2.613	1.820	1.820	1.000	1.000	0.418	0.418
	SSP2	1.916	1.925	1.925	1.324	1.324	1.439	1.439
	SSP3	3.596	2.702	2.702	1.513	1.513	1.029	1.029
	SSP4	3.648	2.392	2.392	1.060	1.060	0.346	0.346
	SSP5	3.675	3.660	3.660	2.640	2.640	1.858	1.858
	SSP1-3.4	2.612	1.555	1.555	0.123	0.123	-0.451	-0.451
	SSP2-3.4	1.680	1.134	1.134	-0.114	-0.114	-0.153	-0.153
	SSP3-3.4	3.532	2.164	2.164	-2.650	-2.650	-0.392	-0.392
	SSP4-3.4	3.510	0.606	0.606	-0.375	-0.375	-1.234	-1.234
	SSP5-3.4	3.586	2.457	2.457	0.623	0.623	-0.189	-0.189

Table S3.6. Scenario setup for France

Period		2019-2020	2021-2025	2026-2030	2031-2035	2036-2040	2041-2045	2046-2050
P	SSP1	0.633	0.618	0.591	0.568	0.541	0.496	0.444
	SSP2	0.585	0.561	0.527	0.492	0.450	0.398	0.348
	SSP3	0.350	0.215	0.106	0.042	-0.036	-0.131	-0.231
	SSP4	0.494	0.430	0.351	0.310	0.252	0.172	0.087
	SSP5	0.823	0.899	0.921	0.902	0.895	0.890	0.889
GDP	SSP1	1.826	1.945	2.182	2.330	2.273	2.123	1.903
	SSP2	1.770	1.770	1.820	1.842	1.830	1.766	1.639
	SSP3	1.498	1.402	1.283	1.139	0.994	0.812	0.587
	SSP4	1.698	1.844	2.062	2.171	2.082	1.925	1.706
	SSP5	2.007	2.391	2.882	3.174	3.141	3.042	2.884
UR	SSP1	0.242	0.213	0.189	0.169	0.151	0.136	0.123
	SSP2	0.242	0.213	0.189	0.169	0.151	0.136	0.123
	SSP3	0.242	0.213	0.189	0.169	0.151	0.136	0.123
	SSP4	0.242	0.213	0.189	0.169	0.151	0.136	0.123
	SSP5	0.242	0.213	0.189	0.169	0.151	0.136	0.123
Primary energy consumption	SSP1	-0.097	-0.319	-0.319	-0.049	-0.049	-0.227	-0.227
	SSP2	0.562	0.436	0.436	0.479	0.479	0.489	0.489
	SSP3	1.130	0.633	0.633	0.377	0.377	0.256	0.256
	SSP4	1.007	0.929	0.929	0.535	0.535	0.200	0.200
	SSP5	0.706	1.592	1.592	1.592	1.592	1.493	1.493
	SSP1-3.4	-0.121	-0.466	-0.466	-0.461	-0.461	-0.469	-0.469
	SSP2-3.4	0.434	0.110	0.110	0.078	0.078	0.069	0.069
	SSP3-3.4	0.726	-0.874	-0.874	-0.250	-0.250	0.498	0.498
	SSP4-3.4	0.725	0.467	0.467	0.063	0.063	-0.059	-0.059
	SSP5-3.4	0.565	1.007	1.007	0.574	0.574	0.953	0.953
Fossil energy consumption	SSP1	-0.322	-0.741	-0.741	-0.375	-0.375	-0.711	-0.711
	SSP2	0.239	0.389	0.389	0.535	0.535	0.529	0.529
	SSP3	1.149	0.578	0.578	0.293	0.293	0.132	0.132
	SSP4	0.731	0.683	0.683	0.276	0.276	-0.013	-0.013
	SSP5	0.604	2.074	2.074	1.935	1.935	1.585	1.585
	SSP1-3.4	-0.324	-0.884	-0.884	-0.958	-0.958	-1.253	-1.253
	SSP2-3.4	0.060	-0.030	-0.030	-0.093	-0.093	-0.371	-0.371
	SSP3-3.4	0.621	-1.584	-1.584	-1.563	-1.563	0.061	0.061
	SSP4-3.4	0.249	-0.308	-0.308	-0.951	-0.951	-1.149	-1.149
	SSP5-3.4	0.424	1.334	1.334	0.661	0.661	0.357	0.357

Table S3.7. Scenario setup for Germany

Period		2019-2020	2021-2025	2026-2030	2031-2035	2036-2040	2041-2045	2046-2050
P	SSP1	0.024	0.032	0.017	0.000	-0.008	-0.021	-0.049
	SSP2	-0.052	-0.055	-0.080	-0.109	-0.136	-0.163	-0.197
	SSP3	-0.270	-0.401	-0.510	-0.585	-0.664	-0.766	-0.881
	SSP4	-0.116	-0.158	-0.222	-0.257	-0.294	-0.348	-0.421
	SSP5	0.178	0.275	0.309	0.310	0.331	0.362	0.387
GDP	SSP1	1.336	1.217	1.226	1.409	1.573	1.436	1.229
	SSP2	1.244	1.005	0.882	1.016	1.269	1.233	1.119
	SSP3	1.022	0.582	0.270	0.235	0.378	0.225	0.003
	SSP4	1.226	1.142	1.136	1.282	1.419	1.268	1.053
	SSP5	1.564	1.715	1.954	2.238	2.411	2.339	2.213
UR	SSP1	0.781	0.590	0.459	0.366	0.297	0.246	0.206
	SSP2	0.478	0.432	0.391	0.355	0.322	0.293	0.267
	SSP3	0.174	0.158	0.144	0.131	0.120	0.110	0.101
	SSP4	0.478	0.432	0.391	0.355	0.322	0.293	0.267
	SSP5	0.781	0.590	0.459	0.366	0.297	0.246	0.206
Primary energy consumption	SSP1	-0.097	-0.319	-0.319	-0.049	-0.049	-0.227	-0.227
	SSP2	0.562	0.436	0.436	0.479	0.479	0.489	0.489
	SSP3	1.130	0.633	0.633	0.377	0.377	0.256	0.256
	SSP4	1.007	0.929	0.929	0.535	0.535	0.200	0.200
	SSP5	0.706	1.592	1.592	1.592	1.592	1.493	1.493
	SSP1-3.4	-0.121	-0.466	-0.466	-0.461	-0.461	-0.469	-0.469
	SSP2-3.4	0.434	0.110	0.110	0.078	0.078	0.069	0.069
	SSP3-3.4	0.726	-0.874	-0.874	-0.250	-0.250	0.498	0.498
	SSP4-3.4	0.725	0.467	0.467	0.063	0.063	-0.059	-0.059
	SSP5-3.4	0.565	1.007	1.007	0.574	0.574	0.953	0.953
Fossil energy consumption	SSP1	-0.322	-0.741	-0.741	-0.375	-0.375	-0.711	-0.711
	SSP2	0.239	0.389	0.389	0.535	0.535	0.529	0.529
	SSP3	1.149	0.578	0.578	0.293	0.293	0.132	0.132
	SSP4	0.731	0.683	0.683	0.276	0.276	-0.013	-0.013
	SSP5	0.604	2.074	2.074	1.935	1.935	1.585	1.585
	SSP1-3.4	-0.324	-0.884	-0.884	-0.958	-0.958	-1.253	-1.253
	SSP2-3.4	0.060	-0.030	-0.030	-0.093	-0.093	-0.371	-0.371
	SSP3-3.4	0.621	-1.584	-1.584	-1.563	-1.563	0.061	0.061
	SSP4-3.4	0.249	-0.308	-0.308	-0.951	-0.951	-1.149	-1.149
	SSP5-3.4	0.424	1.334	1.334	0.661	0.661	0.357	0.357

Table S3.8. Scenario setup for India

Period		2019-2020	2021-2025	2026-2030	2031-2035	2036-2040	2041-2045	2046-2050
P	SSP1	0.957	0.780	0.611	0.499	0.371	0.242	0.106
	SSP2	1.197	1.040	0.898	0.796	0.688	0.585	0.459
	SSP3	1.422	1.326	1.236	1.126	1.061	1.011	0.944
	SSP4	1.096	0.891	0.691	0.566	0.434	0.302	0.150
	SSP5	0.953	0.773	0.605	0.493	0.365	0.237	0.100
GDP	SSP1	6.788	6.840	6.662	6.313	5.638	4.881	4.137
	SSP2	6.802	6.168	5.358	4.753	4.349	3.941	3.566
	SSP3	6.838	5.512	4.206	3.240	2.778	2.372	2.048
	SSP4	6.661	5.832	5.027	4.358	3.854	3.351	2.916
	SSP5	6.893	7.466	7.657	7.367	6.472	5.578	4.748
UR	SSP1	2.652	2.376	2.111	1.861	1.631	1.422	1.236
	SSP2	1.702	1.585	1.472	1.363	1.258	1.159	1.066
	SSP3	0.538	0.532	0.525	0.519	0.512	0.505	0.498
	SSP4	2.652	2.376	2.111	1.861	1.631	1.422	1.236
	SSP5	2.652	2.376	2.111	1.861	1.631	1.422	1.236
Primary energy consumption	SSP1	1.992	1.904	1.904	1.445	1.445	0.784	0.784
	SSP2	2.195	1.915	1.915	1.307	1.307	1.302	1.302
	SSP3	3.284	2.497	2.497	1.437	1.437	1.030	1.030
	SSP4	3.546	2.448	2.448	1.211	1.211	0.515	0.515
	SSP5	3.294	3.194	3.194	2.373	2.373	1.756	1.756
	SSP1-3.4	1.989	1.649	1.649	0.757	0.757	0.247	0.247
	SSP2-3.4	2.083	1.337	1.337	0.627	0.627	0.708	0.708
	SSP3-3.4	3.254	2.188	2.188	-1.524	-1.524	0.671	0.671
	SSP4-3.4	3.452	1.409	1.409	0.653	0.653	0.063	0.063
	SSP5-3.4	3.214	2.212	2.212	0.868	0.868	0.758	0.758
Fossil energy consumption	SSP1	2.613	1.820	1.820	1.000	1.000	0.418	0.418
	SSP2	1.916	1.925	1.925	1.324	1.324	1.439	1.439
	SSP3	3.596	2.702	2.702	1.513	1.513	1.029	1.029
	SSP4	3.648	2.392	2.392	1.060	1.060	0.346	0.346
	SSP5	3.675	3.660	3.660	2.640	2.640	1.858	1.858
	SSP1-3.4	2.612	1.555	1.555	0.123	0.123	-0.451	-0.451
	SSP2-3.4	1.680	1.134	1.134	-0.114	-0.114	-0.153	-0.153
	SSP3-3.4	3.532	2.164	2.164	-2.650	-2.650	-0.392	-0.392
	SSP4-3.4	3.510	0.606	0.606	-0.375	-0.375	-1.234	-1.234
	SSP5-3.4	3.586	2.457	2.457	0.623	0.623	-0.189	-0.189

Table S3.9. Scenario setup for Indonesia

Period		2019-2020	2021-2025	2026-2030	2031-2035	2036-2040	2041-2045	2046-2050
P	SSP1	0.692	0.507	0.338	0.216	0.082	-0.055	-0.198
	SSP2	0.807	0.655	0.510	0.383	0.253	0.115	-0.030
	SSP3	0.930	0.820	0.706	0.554	0.420	0.305	0.200
	SSP4	0.653	0.447	0.257	0.113	-0.043	-0.203	-0.371
	SSP5	0.674	0.480	0.312	0.189	0.056	-0.080	-0.223
GDP	SSP1	6.784	7.065	6.747	6.296	5.468	4.535	3.763
	SSP2	6.695	6.186	5.188	4.437	3.983	3.478	3.031
	SSP3	6.689	5.654	4.216	3.115	2.599	2.087	1.644
	SSP4	6.538	6.004	5.165	4.427	3.901	3.305	2.774
	SSP5	6.885	7.732	7.828	7.378	6.306	5.230	4.330
UR	SSP1	1.847	1.570	1.334	1.135	0.968	0.829	0.713
	SSP2	1.282	1.122	0.983	0.862	0.759	0.670	0.594
	SSP3	0.262	0.266	0.270	0.273	0.277	0.281	0.284
	SSP4	1.847	1.570	1.334	1.135	0.968	0.829	0.713
	SSP5	1.847	1.570	1.334	1.135	0.968	0.829	0.713
Primary energy consumption	SSP1	1.992	1.904	1.904	1.445	1.445	0.784	0.784
	SSP2	2.195	1.915	1.915	1.307	1.307	1.302	1.302
	SSP3	3.284	2.497	2.497	1.437	1.437	1.030	1.030
	SSP4	3.546	2.448	2.448	1.211	1.211	0.515	0.515
	SSP5	3.294	3.194	3.194	2.373	2.373	1.756	1.756
	SSP1-3.4	1.989	1.649	1.649	0.757	0.757	0.247	0.247
	SSP2-3.4	2.083	1.337	1.337	0.627	0.627	0.708	0.708
	SSP3-3.4	3.254	2.188	2.188	-1.524	-1.524	0.671	0.671
	SSP4-3.4	3.452	1.409	1.409	0.653	0.653	0.063	0.063
	SSP5-3.4	3.214	2.212	2.212	0.868	0.868	0.758	0.758
Fossil energy consumption	SSP1	2.613	1.820	1.820	1.000	1.000	0.418	0.418
	SSP2	1.916	1.925	1.925	1.324	1.324	1.439	1.439
	SSP3	3.596	2.702	2.702	1.513	1.513	1.029	1.029
	SSP4	3.648	2.392	2.392	1.060	1.060	0.346	0.346
	SSP5	3.675	3.660	3.660	2.577	2.373	1.756	1.756
	SSP1-3.4	2.612	1.555	1.555	0.123	0.123	-0.451	-0.451
	SSP2-3.4	1.680	1.134	1.134	-0.114	-0.114	-0.153	-0.153
	SSP3-3.4	3.532	2.164	2.164	-2.650	-2.650	-0.392	-0.392
	SSP4-3.4	3.510	0.606	0.606	-0.375	-0.375	-1.234	-1.234
	SSP5-3.4	3.586	2.457	2.457	0.623	0.623	-0.189	-0.189

Table S3.10. Scenario setup for Italy

Period		2019-2020	2021-2025	2026-2030	2031-2035	2036-2040	2041-2045	2046-2050
P	SSP1	0.137	0.114	0.108	0.108	0.099	0.058	-0.005
	SSP2	0.072	0.045	0.037	0.024	-0.002	-0.055	-0.122
	SSP3	-0.152	-0.297	-0.381	-0.432	-0.499	-0.606	-0.742
	SSP4	0.016	-0.045	-0.093	-0.113	-0.143	-0.213	-0.304
	SSP5	0.308	0.370	0.406	0.419	0.428	0.417	0.394
GDP	SSP1	1.274	1.405	1.634	1.769	1.736	1.666	1.571
	SSP2	1.207	1.219	1.244	1.246	1.256	1.287	1.313
	SSP3	0.959	0.794	0.603	0.413	0.292	0.207	0.144
	SSP4	1.172	1.265	1.432	1.513	1.456	1.397	1.339
	SSP5	1.438	1.855	2.363	2.652	2.636	2.607	2.564
UR	SSP1	0.783	0.672	0.579	0.501	0.436	0.381	0.334
	SSP2	0.453	0.426	0.401	0.376	0.353	0.332	0.311
	SSP3	0.179	0.141	0.112	0.090	0.072	0.058	0.047
	SSP4	0.453	0.426	0.401	0.376	0.353	0.332	0.311
	SSP5	0.783	0.672	0.579	0.501	0.436	0.381	0.334
Primary energy consumption	SSP1	-0.097	-0.319	-0.319	-0.049	-0.049	-0.227	-0.227
	SSP2	0.562	0.436	0.436	0.479	0.479	0.489	0.489
	SSP3	1.130	0.633	0.633	0.377	0.377	0.256	0.256
	SSP4	1.007	0.929	0.929	0.535	0.535	0.200	0.200
	SSP5	0.706	1.592	1.592	1.592	1.592	1.493	1.493
	SSP1-3.4	-0.121	-0.466	-0.466	-0.461	-0.461	-0.469	-0.469
	SSP2-3.4	0.434	0.110	0.110	0.078	0.078	0.069	0.069
	SSP3-3.4	0.726	-0.874	-0.874	-0.250	-0.250	0.498	0.498
	SSP4-3.4	0.725	0.467	0.467	0.063	0.063	-0.059	-0.059
	SSP5-3.4	0.565	1.007	1.007	0.574	0.574	0.953	0.953
Fossil energy consumption	SSP1	-0.322	-0.741	-0.741	-0.375	-0.375	-0.711	-0.711
	SSP2	0.239	0.389	0.389	0.535	0.535	0.529	0.529
	SSP3	1.149	0.578	0.578	0.293	0.293	0.132	0.132
	SSP4	0.731	0.683	0.683	0.276	0.276	-0.013	-0.013
	SSP5	0.604	2.074	2.074	1.935	1.935	1.585	1.585
	SSP1-3.4	-0.324	-0.884	-0.884	-0.958	-0.958	-1.253	-1.253
	SSP2-3.4	0.060	-0.030	-0.030	-0.093	-0.093	-0.371	-0.371
	SSP3-3.4	0.621	-1.584	-1.584	-1.563	-1.563	0.061	0.061
	SSP4-3.4	0.249	-0.308	-0.308	-0.951	-0.951	-1.149	-1.149
	SSP5-3.4	0.424	1.334	1.334	0.661	0.661	0.357	0.357

Table S3.11. Scenario setup for Japan

Period		2019-2020	2021-2025	2026-2030	2031-2035	2036-2040	2041-2045	2046-2050
P	SSP1	-0.136	-0.220	-0.281	-0.323	-0.354	-0.381	-0.407
	SSP2	-0.206	-0.316	-0.400	-0.459	-0.501	-0.537	-0.561
	SSP3	-0.376	-0.570	-0.723	-0.824	-0.910	-0.985	-1.061
	SSP4	-0.275	-0.415	-0.531	-0.601	-0.659	-0.721	-0.777
	SSP5	-0.037	-0.073	-0.095	-0.122	-0.132	-0.127	-0.112
GDP	SSP1	1.050	1.362	1.526	1.471	1.266	1.198	1.057
	SSP2	0.942	1.044	0.920	0.659	0.506	0.537	0.507
	SSP3	0.754	0.704	0.430	-0.034	-0.334	-0.429	-0.564
	SSP4	0.923	1.261	1.452	1.362	1.118	1.028	0.873
	SSP5	1.200	1.829	2.262	2.341	2.100	2.037	1.930
UR	SSP1	0.865	0.724	0.610	0.519	0.445	0.384	0.333
	SSP2	0.540	0.500	0.463	0.428	0.396	0.367	0.339
	SSP3	0.194	0.147	0.112	0.085	0.066	0.051	0.039
	SSP4	0.540	0.500	0.463	0.428	0.396	0.367	0.339
	SSP5	0.865	0.724	0.610	0.519	0.445	0.384	0.333
Primary energy consumption	SSP1	-0.097	-0.319	-0.319	-0.049	-0.049	-0.227	-0.227
	SSP2	0.562	0.436	0.436	0.479	0.479	0.489	0.489
	SSP3	1.130	0.633	0.633	0.377	0.377	0.256	0.256
	SSP4	1.007	0.929	0.929	0.535	0.535	0.200	0.200
	SSP5	0.706	1.592	1.592	1.592	1.592	1.493	1.493
	SSP1-3.4	-0.121	-0.466	-0.466	-0.461	-0.461	-0.469	-0.469
	SSP2-3.4	0.434	0.110	0.110	0.078	0.078	0.069	0.069
	SSP3-3.4	0.726	-0.874	-0.874	-0.250	-0.250	0.498	0.498
	SSP4-3.4	0.725	0.467	0.467	0.063	0.063	-0.059	-0.059
	SSP5-3.4	0.565	1.007	1.007	0.574	0.574	0.953	0.953
Fossil energy consumption	SSP1	-0.322	-0.741	-0.741	-0.375	-0.375	-0.711	-0.711
	SSP2	0.239	0.389	0.389	0.535	0.535	0.529	0.529
	SSP3	1.149	0.578	0.578	0.293	0.293	0.132	0.132
	SSP4	0.731	0.683	0.683	0.276	0.276	-0.013	-0.013
	SSP5	0.604	2.074	2.074	1.935	1.935	1.585	1.585
	SSP1-3.4	-0.324	-0.884	-0.884	-0.958	-0.958	-1.253	-1.253
	SSP2-3.4	0.060	-0.030	-0.030	-0.093	-0.093	-0.371	-0.371
	SSP3-3.4	0.621	-1.584	-1.584	-1.563	-1.563	0.061	0.061
	SSP4-3.4	0.249	-0.308	-0.308	-0.951	-0.951	-1.149	-1.149
	SSP5-3.4	0.424	1.334	1.334	0.661	0.661	0.357	0.357

Table S3.12. Scenario setup for Korea, Rep.

Period		2019-2020	2021-2025	2026-2030	2031-2035	2036-2040	2041-2045	2046-2050
P	SSP1	0.252	0.181	0.096	-0.014	-0.137	-0.258	-0.374
	SSP2	0.192	0.106	0.008	-0.129	-0.290	-0.444	-0.586
	SSP3	0.067	-0.098	-0.273	-0.450	-0.655	-0.874	-1.087
	SSP4	0.131	0.008	-0.127	-0.260	-0.420	-0.591	-0.763
	SSP5	0.337	0.306	0.256	0.152	0.039	-0.056	-0.131
GDP	SSP1	3.875	3.459	2.894	2.369	1.851	1.453	1.107
	SSP2	3.750	3.178	2.541	2.021	1.611	1.314	1.042
	SSP3	3.532	2.759	2.008	1.343	0.856	0.470	0.106
	SSP4	3.748	3.369	2.818	2.252	1.702	1.302	0.951
	SSP5	4.063	3.922	3.455	2.942	2.416	2.089	1.834
UR	SSP1	0.306	0.286	0.266	0.247	0.227	0.209	0.191
	SSP2	0.306	0.286	0.266	0.247	0.227	0.209	0.191
	SSP3	0.306	0.286	0.266	0.247	0.227	0.209	0.191
	SSP4	0.306	0.286	0.266	0.247	0.227	0.209	0.191
	SSP5	0.306	0.286	0.266	0.247	0.227	0.209	0.191
Primary energy consumption	SSP1	1.992	1.904	1.904	1.445	1.445	0.784	0.784
	SSP2	2.195	1.915	1.915	1.307	1.307	1.302	1.302
	SSP3	3.284	2.497	2.497	1.437	1.437	1.030	1.030
	SSP4	3.546	2.448	2.448	1.211	1.211	0.515	0.515
	SSP5	3.294	3.194	3.194	2.373	2.373	1.756	1.756
	SSP1-3.4	1.989	1.649	1.649	0.757	0.757	0.247	0.247
	SSP2-3.4	2.083	1.337	1.337	0.627	0.627	0.708	0.708
	SSP3-3.4	3.254	2.188	2.188	-1.524	-1.524	0.671	0.671
	SSP4-3.4	3.452	1.409	1.409	0.653	0.653	0.063	0.063
	SSP5-3.4	3.214	2.212	2.212	0.868	0.868	0.758	0.758
Fossil energy consumption	SSP1	2.613	1.820	1.820	1.000	1.000	0.418	0.418
	SSP2	1.916	1.925	1.925	1.324	1.324	1.439	1.439
	SSP3	3.596	2.702	2.702	1.513	1.513	1.029	1.029
	SSP4	3.648	2.392	2.392	1.060	1.060	0.346	0.346
	SSP5	3.675	3.660	3.660	2.640	2.640	1.858	1.858
	SSP1-3.4	2.612	1.555	1.555	0.123	0.123	-0.451	-0.451
	SSP2-3.4	1.680	1.134	1.134	-0.114	-0.114	-0.153	-0.153
	SSP3-3.4	3.532	2.164	2.164	-2.650	-2.650	-0.392	-0.392
	SSP4-3.4	3.510	0.606	0.606	-0.375	-0.375	-1.234	-1.234
	SSP5-3.4	3.586	2.457	2.457	0.623	0.623	-0.189	-0.189

Table S3.13. Scenario setup for Mexico

Period		2019-2020	2021-2025	2026-2030	2031-2035	2036-2040	2041-2045	2046-2050
P	SSP1	0.815	0.624	0.441	0.306	0.165	0.023	-0.113
	SSP2	1.014	0.877	0.738	0.601	0.477	0.358	0.247
	SSP3	1.292	1.269	1.187	1.056	0.959	0.888	0.823
	SSP4	0.882	0.691	0.497	0.348	0.194	0.041	-0.108
	SSP5	0.705	0.456	0.269	0.130	-0.014	-0.160	-0.300
GDP	SSP1	3.170	3.306	3.513	3.528	3.223	2.929	2.646
	SSP2	3.314	3.166	2.982	2.774	2.576	2.463	2.396
	SSP3	3.507	3.091	2.607	2.188	1.853	1.621	1.510
	SSP4	3.161	2.938	2.907	2.719	2.422	2.192	2.065
	SSP5	3.112	3.538	4.117	4.277	3.848	3.436	3.091
UR	SSP1	0.747	0.590	0.471	0.380	0.309	0.253	0.209
	SSP2	0.403	0.361	0.325	0.293	0.264	0.239	0.217
	SSP3	0.202	0.219	0.236	0.251	0.264	0.275	0.282
	SSP4	0.747	0.590	0.471	0.380	0.309	0.253	0.209
	SSP5	0.747	0.590	0.471	0.380	0.309	0.253	0.209
Primary energy consumption	SSP1	2.473	1.654	1.654	1.710	1.710	1.298	1.298
	SSP2	2.139	1.897	1.897	1.820	1.820	1.249	1.249
	SSP3	2.532	2.154	2.154	1.493	1.493	1.231	1.231
	SSP4	2.967	2.301	2.301	1.477	1.477	0.935	0.935
	SSP5	3.098	2.345	2.345	1.895	1.895	1.722	1.722
	SSP1-3.4	2.476	1.421	1.421	1.161	1.161	0.928	0.928
	SSP2-3.4	1.933	1.629	1.629	1.704	1.704	1.188	1.188
	SSP3-3.4	2.541	2.123	2.123	-0.067	-0.067	0.997	0.997
	SSP4-3.4	2.491	1.953	1.953	1.209	1.209	0.712	0.712
	SSP5-3.4	3.098	1.911	1.911	0.713	0.713	1.168	1.168
Fossil energy consumption	SSP1	3.006	1.745	1.745	1.149	1.149	0.970	0.970
	SSP2	2.111	2.293	2.293	2.130	2.130	1.088	1.088
	SSP3	2.636	2.289	2.289	1.515	1.515	1.134	1.134
	SSP4	3.096	2.229	2.229	1.320	1.320	0.769	0.769
	SSP5	3.242	3.391	2.997	1.895	1.895	1.688	1.688
	SSP1-3.4	3.048	1.431	1.431	0.330	0.330	0.214	0.214
	SSP2-3.4	1.725	1.276	1.276	1.314	1.314	0.755	0.755
	SSP3-3.4	2.610	2.170	2.170	-1.046	-1.046	-0.138	-0.138
	SSP4-3.4	2.305	1.533	1.533	0.719	0.719	-0.029	-0.029
	SSP5-3.4	3.224	2.758	2.757	0.713	0.713	0.088	0.088

Table S3.14. Scenario setup for Russian Federation

Period		2019-2020	2021-2025	2026-2030	2031-2035	2036-2040	2041-2045	2046-2050
P	SSP1	-0.157	-0.224	-0.268	-0.240	-0.226	-0.256	-0.323
	SSP2	-0.099	-0.150	-0.182	-0.164	-0.101	-0.081	-0.076
	SSP3	-0.118	-0.230	-0.271	-0.261	-0.179	-0.098	-0.042
	SSP4	-0.195	-0.275	-0.336	-0.341	-0.330	-0.366	-0.412
	SSP5	-0.081	-0.083	-0.103	-0.064	-0.051	-0.090	-0.166
GDP	SSP1	3.775	3.746	3.820	3.401	2.589	1.767	1.106
	SSP2	3.658	3.292	2.950	2.462	1.989	1.482	1.127
	SSP3	3.540	2.874	2.383	1.910	1.438	0.803	0.336
	SSP4	3.570	3.290	3.142	2.822	2.277	1.585	1.051
	SSP5	3.948	4.631	4.946	4.564	3.567	2.602	1.864
UR	SSP1	0.785	0.587	0.453	0.359	0.290	0.239	0.200
	SSP2	0.498	0.445	0.399	0.359	0.323	0.292	0.264
	SSP3	0.172	0.162	0.153	0.144	0.136	0.129	0.122
	SSP4	0.785	0.587	0.453	0.359	0.290	0.239	0.200
	SSP5	0.785	0.587	0.453	0.359	0.290	0.239	0.200
Primary energy consumption	SSP1	0.456	0.890	0.890	0.129	0.129	-0.653	-0.653
	SSP2	0.517	0.399	0.399	0.533	0.533	0.905	0.905
	SSP3	1.654	1.393	1.393	0.861	0.861	0.177	0.177
	SSP4	1.341	0.862	0.862	0.328	0.328	-0.185	-0.185
	SSP5	0.994	1.237	1.237	1.432	1.432	0.881	0.881
	SSP1-3.4	0.455	0.708	0.708	-0.269	-0.269	-0.886	-0.886
	SSP2-3.4	0.511	-0.120	-0.120	-0.170	-0.170	0.196	0.196
	SSP3-3.4	1.688	1.462	1.462	-1.725	-1.725	0.295	0.295
	SSP4-3.4	1.208	0.136	0.136	-0.129	-0.129	-0.626	-0.626
	SSP5-3.4	0.705	0.194	0.194	-0.589	-0.589	-0.341	-0.341
Fossil energy consumption	SSP1	0.314	0.732	0.732	0.065	0.065	-0.702	-0.702
	SSP2	0.246	0.412	0.412	0.764	0.764	0.952	0.952
	SSP3	1.625	1.355	1.355	0.886	0.886	0.203	0.203
	SSP4	1.028	0.536	0.536	0.004	0.004	-0.479	-0.479
	SSP5	0.880	1.344	1.344	1.609	1.609	1.031	1.031
	SSP1-3.4	0.312	0.429	0.429	-0.499	-0.499	-0.997	-0.997
	SSP2-3.4	0.234	-0.236	-0.236	-0.413	-0.413	0.051	0.051
	SSP3-3.4	1.660	1.427	1.427	-2.866	-2.866	-1.306	-1.306
	SSP4-3.4	0.862	-0.738	-0.738	-1.126	-1.126	-1.924	-1.924
	SSP5-3.4	0.565	0.164	0.164	-0.860	-0.860	-0.686	-0.686

Table S3.15. Scenario setup for Saudi Arabia

Period		2019-2020	2021-2025	2026-2030	2031-2035	2036-2040	2041-2045	2046-2050
P	SSP1	2.084	1.779	1.524	1.359	1.173	0.958	0.739
	SSP2	2.257	1.987	1.774	1.595	1.417	1.221	1.048
	SSP3	2.248	1.957	1.818	1.664	1.542	1.434	1.335
	SSP4	2.487	2.293	2.117	1.915	1.755	1.626	1.522
	SSP5	2.32	2.089	1.794	1.595	1.39	1.156	0.917
GDP	SSP1	4.146	4.039	4.07	3.357	2.834	2.377	1.981
	SSP2	4.26	3.977	3.813	2.976	2.687	2.307	1.956
	SSP3	4.249	3.953	3.996	3.378	2.925	2.254	1.554
	SSP4	4.526	4.424	4.688	4.225	3.748	3.132	2.545
	SSP5	4.394	4.698	5.239	4.917	4.36	3.632	2.93
UR	SSP1	0.319	0.293	0.269	0.246	0.225	0.205	0.188
	SSP2	0.319	0.293	0.269	0.246	0.225	0.205	0.188
	SSP3	0.319	0.293	0.269	0.246	0.225	0.205	0.188
	SSP4	0.319	0.293	0.269	0.246	0.225	0.205	0.188
	SSP5	0.319	0.293	0.269	0.246	0.225	0.205	0.188
Primary energy consumption	SSP1	1.335	2.054	2.054	2.771	2.771	2.543	2.543
	SSP2	2.416	2.692	2.692	2.403	2.403	2.08	2.08
	SSP3	3.185	2.963	2.963	2.421	2.421	1.996	1.996
	SSP4	2.672	2.835	2.835	2.047	2.047	1.577	1.577
	SSP5	4.058	2.195	2.195	3.008	3.008	2.978	2.978
	SSP1-3.4	1.33	1.847	1.847	2.184	2.184	1.961	1.961
	SSP2-3.4	2.399	2.006	2.006	1.964	1.964	1.915	1.915
	SSP3-3.4	3.216	2.983	2.983	0.164	0.164	1.388	1.388
	SSP4-3.4	2.403	2.412	2.412	1.716	1.716	1.275	1.275
	SSP5-3.4	3.918	1.225	1.225	1.591	1.591	2.259	2.259

Table S3.16. Scenario setup for South Africa

Period		2019-2020	2021-2025	2026-2030	2031-2035	2036-2040	2041-2045	2046-2050
P	SSP1	0.868	0.747	0.586	0.483	0.378	0.267	0.158
	SSP2	0.863	0.737	0.605	0.488	0.405	0.323	0.254
	SSP3	0.665	0.570	0.514	0.439	0.393	0.362	0.367
	SSP4	0.713	0.508	0.334	0.174	0.043	-0.087	-0.203
	SSP5	0.967	0.890	0.721	0.611	0.506	0.392	0.275
GDP	SSP1	4.504	4.443	4.235	3.979	3.547	3.054	2.567
	SSP2	4.418	4.000	3.490	3.090	2.808	2.525	2.275
	SSP3	4.144	3.379	2.569	2.017	1.711	1.457	1.298
	SSP4	4.244	3.698	3.249	2.806	2.452	2.107	1.799
	SSP5	4.618	4.857	4.952	4.833	4.367	3.840	3.319
UR	SSP1	1.042	0.935	0.834	0.740	0.655	0.576	0.506
	SSP2	0.785	0.677	0.588	0.513	0.450	0.397	0.352
	SSP3	0.290	0.259	0.231	0.207	0.186	0.167	0.151
	SSP4	1.042	0.935	0.834	0.740	0.655	0.576	0.506
	SSP5	1.042	0.935	0.834	0.740	0.655	0.576	0.506
Primary energy consumption	SSP1	1.335	2.054	2.054	2.771	2.771	2.543	2.543
	SSP2	2.416	2.692	2.692	2.403	2.403	2.080	2.080
	SSP3	3.185	2.963	2.963	2.421	2.421	1.996	1.996
	SSP4	2.672	2.835	2.835	2.047	2.047	1.577	1.577
	SSP5	4.058	2.195	2.195	3.008	3.008	2.978	2.978
	SSP1-3.4	1.330	1.847	1.847	2.184	2.184	1.961	1.961
	SSP2-3.4	2.399	2.006	2.006	1.964	1.964	1.915	1.915
	SSP3-3.4	3.216	2.983	2.983	0.164	0.164	1.388	1.388
	SSP4-3.4	2.403	2.412	2.412	1.716	1.716	1.275	1.275
	SSP5-3.4	3.918	1.225	1.225	1.591	1.591	2.259	2.259
Fossil energy consumption	SSP1	2.230	2.098	2.098	2.334	2.334	2.000	2.000
	SSP2	2.973	2.985	2.985	2.501	2.501	1.866	1.866
	SSP3	3.351	3.151	3.151	2.533	2.533	2.033	2.033
	SSP4	2.407	2.857	2.857	1.892	1.892	1.391	1.391
	SSP5	5.071	2.962	2.195	3.008	3.008	2.978	2.978
	SSP1-3.4	2.225	1.874	1.874	1.626	1.626	1.023	1.023
	SSP2-3.4	2.950	1.936	1.936	1.587	1.587	1.414	1.414
	SSP3-3.4	3.388	3.152	3.152	-0.622	-0.622	0.485	0.485
	SSP4-3.4	2.009	1.951	1.951	1.172	1.172	0.570	0.570
	SSP5-3.4	4.900	1.832	1.383	1.591	1.591	1.861	1.861

Table S3.17. Scenario setup for Turkey

Period		2019-2020	2021-2025	2026-2030	2031-2035	2036-2040	2041-2045	2046-2050
P	SSP1	0.815	0.636	0.474	0.368	0.248	0.112	-0.029
	SSP2	1.010	0.873	0.736	0.610	0.497	0.386	0.279
	SSP3	1.268	1.182	1.073	0.937	0.847	0.805	0.767
	SSP4	0.999	0.817	0.619	0.478	0.342	0.207	0.068
	SSP5	0.810	0.629	0.468	0.364	0.247	0.111	-0.029
GDP	SSP1	4.206	3.921	3.708	3.434	3.075	2.631	2.189
	SSP2	4.297	3.655	3.164	2.806	2.629	2.403	2.196
	SSP3	4.410	3.284	2.491	1.993	1.793	1.583	1.428
	SSP4	4.238	3.367	2.879	2.460	2.209	1.895	1.636
	SSP5	4.252	4.262	4.307	4.133	3.713	3.236	2.779
UR	SSP1	0.743	0.634	0.545	0.470	0.408	0.356	0.312
	SSP2	0.398	0.380	0.362	0.344	0.327	0.311	0.294
	SSP3	0.101	0.086	0.073	0.062	0.053	0.045	0.039
	SSP4	0.743	0.634	0.545	0.470	0.408	0.356	0.312
	SSP5	0.743	0.634	0.545	0.470	0.408	0.356	0.312
Primary energy consumption	SSP1	-0.097	-0.319	-0.319	-0.049	-0.049	-0.227	-0.227
	SSP2	0.562	0.436	0.436	0.479	0.479	0.489	0.489
	SSP3	1.130	0.633	0.633	0.377	0.377	0.256	0.256
	SSP4	1.007	0.929	0.929	0.535	0.535	0.200	0.200
	SSP5	0.706	1.592	1.592	1.592	1.592	1.493	1.493
	SSP1-3.4	-0.121	-0.466	-0.466	-0.461	-0.461	-0.469	-0.469
	SSP2-3.4	0.434	0.110	0.110	0.078	0.078	0.069	0.069
	SSP3-3.4	0.726	-0.874	-0.874	-0.250	-0.250	0.498	0.498
	SSP4-3.4	0.725	0.467	0.467	0.063	0.063	-0.059	-0.059
	SSP5-3.4	0.565	1.007	1.007	0.574	0.574	0.953	0.953
Fossil energy consumption	SSP1	-0.322	-0.741	-0.741	-0.375	-0.375	-0.711	-0.711
	SSP2	0.239	0.389	0.389	0.535	0.535	0.529	0.529
	SSP3	1.149	0.578	0.578	0.293	0.293	0.132	0.132
	SSP4	0.731	0.683	0.683	0.276	0.276	-0.013	-0.013
	SSP5	0.604	2.074	2.074	1.935	1.935	1.585	1.585
	SSP1-3.4	-0.324	-0.884	-0.884	-0.958	-0.958	-1.253	-1.253
	SSP2-3.4	0.060	-0.030	-0.030	-0.093	-0.093	-0.371	-0.371
	SSP3-3.4	0.621	-1.584	-1.584	-1.563	-1.563	0.061	0.061
	SSP4-3.4	0.249	-0.308	-0.308	-0.951	-0.951	-1.149	-1.149
	SSP5-3.4	0.424	1.334	1.334	0.661	0.661	0.357	0.357

Table S3.18. Scenario setup for United Kingdom

Period		2019-2020	2021-2025	2026-2030	2031-2035	2036-2040	2041-2045	2046-2050
P	SSP1	0.663	0.638	0.590	0.551	0.531	0.514	0.485
	SSP2	0.625	0.592	0.536	0.484	0.455	0.440	0.414
	SSP3	0.365	0.208	0.071	-0.015	-0.077	-0.135	-0.212
	SSP4	0.519	0.437	0.332	0.275	0.228	0.182	0.117
	SSP5	0.873	0.946	0.950	0.922	0.927	0.949	0.963
GDP	SSP1	2.558	2.330	2.263	2.234	2.159	2.039	1.788
	SSP2	2.466	2.050	1.810	1.715	1.751	1.751	1.623
	SSP3	2.140	1.599	1.191	0.921	0.821	0.701	0.477
	SSP4	2.388	2.220	2.102	2.008	1.881	1.735	1.497
	SSP5	2.820	2.882	3.040	3.107	3.027	2.949	2.770
UR	SSP1	0.674	0.586	0.499	0.417	0.343	0.278	0.223
	SSP2	0.416	0.343	0.288	0.244	0.210	0.182	0.159
	SSP3	0.276	0.275	0.272	0.267	0.261	0.252	0.242
	SSP4	0.416	0.343	0.288	0.244	0.210	0.182	0.159
	SSP5	0.674	0.586	0.499	0.417	0.343	0.278	0.223
Primary energy consumption	SSP1	-0.097	-0.319	-0.319	-0.049	-0.049	-0.227	-0.227
	SSP2	0.562	0.436	0.436	0.479	0.479	0.489	0.489
	SSP3	1.130	0.633	0.633	0.377	0.377	0.256	0.256
	SSP4	1.007	0.929	0.929	0.535	0.535	0.200	0.200
	SSP5	0.706	1.592	1.592	1.592	1.592	1.493	1.493
	SSP1-3.4	-0.121	-0.466	-0.466	-0.461	-0.461	-0.469	-0.469
	SSP2-3.4	0.434	0.110	0.110	0.078	0.078	0.069	0.069
	SSP3-3.4	0.726	-0.874	-0.874	-0.250	-0.250	0.498	0.498
	SSP4-3.4	0.725	0.467	0.467	0.063	0.063	-0.059	-0.059
	SSP5-3.4	0.565	1.007	1.007	0.574	0.574	0.953	0.953
Fossil energy consumption	SSP1	-0.322	-0.741	-0.741	-0.375	-0.375	-0.711	-0.711
	SSP2	0.239	0.389	0.389	0.535	0.535	0.529	0.529
	SSP3	1.149	0.578	0.578	0.293	0.293	0.132	0.132
	SSP4	0.731	0.683	0.683	0.276	0.276	-0.013	-0.013
	SSP5	0.604	2.074	2.074	1.935	1.935	1.585	1.585
	SSP1-3.4	-0.324	-0.884	-0.884	-0.958	-0.958	-1.253	-1.253
	SSP2-3.4	0.060	-0.030	-0.030	-0.093	-0.093	-0.371	-0.371
	SSP3-3.4	0.621	-1.584	-1.584	-1.563	-1.563	0.061	0.061
	SSP4-3.4	0.249	-0.308	-0.308	-0.951	-0.951	-1.149	-1.149
	SSP5-3.4	0.424	1.334	1.334	0.661	0.661	0.357	0.357

Table S3.19. Scenario setup for United States

Period		2019-2020	2021-2025	2026-2030	2031-2035	2036-2040	2041-2045	2046-2050
P	SSP1	0.822	0.800	0.746	0.696	0.644	0.582	0.535
	SSP2	0.788	0.759	0.698	0.630	0.567	0.506	0.468
	SSP3	0.505	0.331	0.185	0.092	0.001	-0.097	-0.181
	SSP4	0.706	0.641	0.534	0.467	0.389	0.303	0.230
	SSP5	1.069	1.162	1.155	1.103	1.068	1.045	1.047
GDP	SSP1	3.037	2.632	2.385	2.135	1.854	1.601	1.354
	SSP2	2.913	2.304	1.896	1.592	1.399	1.236	1.075
	SSP3	2.580	1.833	1.338	1.014	0.807	0.633	0.418
	SSP4	2.887	2.550	2.293	2.015	1.686	1.412	1.172
	SSP5	3.338	3.223	3.170	2.987	2.690	2.499	2.341
UR	SSP1	0.318	0.292	0.268	0.246	0.225	0.205	0.187
	SSP2	0.318	0.292	0.268	0.246	0.225	0.205	0.187
	SSP3	0.318	0.292	0.268	0.246	0.225	0.205	0.187
	SSP4	0.318	0.292	0.268	0.246	0.225	0.205	0.187
	SSP5	0.318	0.292	0.268	0.246	0.225	0.205	0.187
Primary energy consumption	SSP1	-0.097	-0.319	-0.319	-0.049	-0.049	-0.227	-0.227
	SSP2	0.562	0.436	0.436	0.479	0.479	0.489	0.489
	SSP3	1.130	0.633	0.633	0.377	0.377	0.256	0.256
	SSP4	1.007	0.929	0.929	0.535	0.535	0.200	0.200
	SSP5	0.706	1.592	1.592	1.592	1.592	1.493	1.493
	SSP1-3.4	-0.121	-0.466	-0.466	-0.461	-0.461	-0.469	-0.469
	SSP2-3.4	0.434	0.110	0.110	0.078	0.078	0.069	0.069
	SSP3-3.4	0.726	-0.874	-0.874	-0.250	-0.250	0.498	0.498
	SSP4-3.4	0.725	0.467	0.467	0.063	0.063	-0.059	-0.059
	SSP5-3.4	0.565	1.007	1.007	0.574	0.574	0.953	0.953
Fossil energy consumption	SSP1	-0.322	-0.741	-0.741	-0.375	-0.375	-0.711	-0.711
	SSP2	0.239	0.389	0.389	0.535	0.535	0.529	0.529
	SSP3	1.149	0.578	0.578	0.293	0.293	0.132	0.132
	SSP4	0.731	0.683	0.683	0.276	0.276	-0.013	-0.013
	SSP5	0.604	2.074	2.074	1.935	1.935	1.585	1.585
	SSP1-3.4	-0.324	-0.884	-0.884	-0.958	-0.958	-1.253	-1.253
	SSP2-3.4	0.060	-0.030	-0.030	-0.093	-0.093	-0.371	-0.371
	SSP3-3.4	0.621	-1.584	-1.584	-1.563	-1.563	0.061	0.061
	SSP4-3.4	0.249	-0.308	-0.308	-0.951	-0.951	-1.149	-1.149
	SSP5-3.4	0.424	1.334	1.334	0.661	0.661	0.357	0.357

Table S3.20. Scenario setup for the World

Period		2019-2020	2021-2025	2026-2030	2031-2035	2036-2040	2041-2045	2046-2050
P	SSP1	0.838	0.696	0.549	0.450	0.341	0.224	0.104
	SSP2	0.991	0.880	0.766	0.664	0.568	0.472	0.373
	SSP3	1.121	1.052	0.978	0.877	0.808	0.758	0.710
	SSP4	0.975	0.860	0.744	0.645	0.555	0.470	0.385
	SSP5	0.853	0.718	0.578	0.481	0.375	0.265	0.155
GDP	SSP1	4.391	4.313	4.178	3.870	3.398	2.947	2.505
	SSP2	4.316	3.804	3.262	2.793	2.539	2.324	2.109
	SSP3	4.185	3.349	2.541	1.881	1.568	1.305	1.056
	SSP4	4.251	3.775	3.310	2.800	2.394	2.027	1.680
	SSP5	4.581	4.957	5.108	4.829	4.194	3.652	3.166
UR	SSP1	1.380	1.230	1.097	0.974	0.865	0.768	0.680
	SSP2	0.867	0.790	0.721	0.659	0.604	0.555	0.513
	SSP3	0.257	0.215	0.183	0.166	0.150	0.135	0.126
	SSP4	1.320	1.181	1.054	0.943	0.841	0.749	0.667
	SSP5	1.394	1.247	1.112	0.985	0.873	0.774	0.685
Primary energy consumption	SSP1	1.042	1.080	1.080	1.092	1.092	0.720	0.720
	SSP2	1.483	1.397	1.397	1.193	1.193	1.157	1.157
	SSP3	2.214	1.747	1.747	1.160	1.160	0.871	0.871
	SSP4	2.288	1.871	1.871	1.082	1.082	0.576	0.576
	SSP5	2.231	2.336	2.336	2.137	2.137	1.835	1.835
	SSP1-3.4	1.032	0.872	0.872	0.524	0.524	0.278	0.278
	SSP2-3.4	1.380	0.922	0.922	0.683	0.683	0.728	0.728
	SSP3-3.4	2.009	1.152	1.152	-0.915	-0.915	0.714	0.714
	SSP4-3.4	2.077	1.172	1.172	0.611	0.611	0.231	0.231
	SSP5-3.4	2.116	1.532	1.532	0.807	0.807	1.059	1.059
Fossil energy consumption	SSP1	1.263	0.911	0.911	0.709	0.709	0.318	0.318
	SSP2	1.261	1.418	1.418	1.251	1.251	1.165	1.165
	SSP3	2.287	1.781	1.781	1.149	1.149	0.808	0.808
	SSP4	2.142	1.713	1.713	0.865	0.865	0.363	0.363
	SSP5	2.406	2.866	2.866	2.503	2.503	1.937	1.937
	SSP1-3.4	1.264	0.682	0.682	-0.039	-0.039	-0.443	-0.443
	SSP2-3.4	1.085	0.727	0.727	0.229	0.229	0.131	0.131
	SSP3-3.4	2.008	0.882	0.882	-2.071	-2.071	-0.166	-0.166
	SSP4-3.4	1.808	0.408	0.408	-0.327	-0.327	-0.879	-0.879
	SSP5-3.4	2.265	1.879	1.879	0.809	0.809	0.352	0.352
